# Defining the diverse spectrum of inversions, complex structural variation, and chromothripsis in the morbid human genome

**DOI:** 10.1186/s13059-017-1158-6

**Published:** 2017-03-06

**Authors:** Ryan L. Collins, Harrison Brand, Claire E. Redin, Carrie Hanscom, Caroline Antolik, Matthew R. Stone, Joseph T. Glessner, Tamara Mason, Giulia Pregno, Naghmeh Dorrani, Giorgia Mandrile, Daniela Giachino, Danielle Perrin, Cole Walsh, Michelle Cipicchio, Maura Costello, Alexei Stortchevoi, Joon-Yong An, Benjamin B. Currall, Catarina M. Seabra, Ashok Ragavendran, Lauren Margolin, Julian A. Martinez-Agosto, Diane Lucente, Brynn Levy, Stephan J. Sanders, Ronald J. Wapner, Fabiola Quintero-Rivera, Wigard Kloosterman, Michael E. Talkowski

**Affiliations:** 1 Molecular Neurogenetics Unit and Psychiatric and Neurodevelopmental Genetics Unit, Center for Genomic Medicine, and Department of Neurology, Massachusetts General Hospital, Boston, MA 02114 USA; 2 Program in Bioinformatics and Integrative Genomics, Division of Medical Sciences, Harvard Medical School, Boston, MA 02115 USA; 3 Program in Population and Medical Genetics and Genomics Platform, The Broad Institute of M.I.T. and Harvard, Cambridge, MA 02142 USA; 4 Medical Genetics Unit, Department of Clinical and Biological Sciences, University of Torino, Orbassano, Italy; 5 Department of Pathology & Laboratory Medicine and UCLA Clinical Genomics Center, David Geffen School of Medicine, University of California Los Angeles, UCLA, Los Angeles, CA 90095 USA; 6 Department of Psychiatry, University of California San Francisco, San Francisco, CA 94103 USA; 7 GABBA Program, University of Porto, Porto, 4099-002 Portugal; 8 Department of Pathology, Columbia University, New York, NY 10032 USA; 9 Division of Maternal-Fetal Medicine, Department of Obstetrics and Gynecology, Columbia University Medical Center, New York, NY 10032 USA; 10 Department of Medical Genetics, Center for Molecular Medicine, University Medical Center Utrecht, Utrecht, 3584CG The Netherlands

**Keywords:** Structural variation, Inversion, Complex chromosomal rearrangement, Chromoanagenesis, Chromothripsis, Autism, Neurodevelopmental disorders, Copynumber variation, Whole-genome sequencing, Germline mutation

## Abstract

**Background:**

Structural variation (SV) influences genome organization and contributes to human disease. However, the complete mutational spectrum of SV has not been routinely captured in disease association studies.

**Results:**

We sequenced 689 participants with autism spectrum disorder (ASD) and other developmental abnormalities to construct a genome-wide map of large SV. Using long-insert jumping libraries at 105X mean physical coverage and linked-read whole-genome sequencing from 10X Genomics, we document seven major SV classes at ~5 kb SV resolution. Our results encompass 11,735 distinct large SV sites, 38.1% of which are novel and 16.8% of which are balanced or complex. We characterize 16 recurrent subclasses of complex SV (cxSV), revealing that: (1) cxSV are larger and rarer than canonical SV; (2) each genome harbors 14 large cxSV on average; (3) 84.4% of large cxSVs involve inversion; and (4) most large cxSV (93.8%) have not been delineated in previous studies. Rare SVs are more likely to disrupt coding and regulatory non-coding loci, particularly when truncating constrained and disease-associated genes. We also identify multiple cases of catastrophic chromosomal rearrangements known as chromoanagenesis, including somatic chromoanasynthesis, and extreme balanced germline chromothripsis events involving up to 65 breakpoints and 60.6 Mb across four chromosomes, further defining rare categories of extreme cxSV.

**Conclusions:**

These data provide a foundational map of large SV in the morbid human genome and demonstrate a previously underappreciated abundance and diversity of cxSV that should be considered in genomic studies of human disease.

**Electronic supplementary material:**

The online version of this article (doi:10.1186/s13059-017-1158-6) contains supplementary material, which is available to authorized users.

## Background

Structural variation (SV), or the rearrangement of chromosomal segments (≥50 bp), is a major driver of the organization and content of individual genomes [1]. SV manifests in multiple mutational forms, canonically categorized as “balanced” SV—rearrangements lacking major gain or loss of genomic DNA, such as inversions, multiple classes of insertions, and translocations—and “unbalanced” SV, or copy number variants (CNV), which involve changes in DNA dosage [2, 3]. Recent research has demonstrated that some rearrangements have multiple, compounded mutational signatures and do not fit into a single canonical SV category [4–9]. These non-canonical, complex SVs (cxSV) span a heterogeneous range from relatively simple CNV-flanked inversions to extreme rearrangements involving dozens of loci across multiple chromosomes [4, 10]. The most severe cxSVs are thought to involve sudden chromosome pulverization and reorganization; this group of ultra-rare, catastrophic cxSVs are known collectively as chromoanagenesis [11], which encompasses three core proposed mechanisms: chromothripsis [12]; chromoanasynthesis [13]; and chromoplexy [14]. The most commonly reported of these, chromothripsis, was first observed in cancer with interspersed deletion bridges between fragments of derivative chromosomes [12, 15, 16], while subsequent studies discovered both balanced and unbalanced forms of chromothripsis in the human germline [9, 10, 17, 18]. Though less frequently reported, chromoanasynthesis and chromoplexy have also been observed in the human germline [9, 13, 19–23]. Despite these discoveries, the patterns, rates, and properties of cxSVs have primarily been the focus of cancer genomics and such rearrangements remain largely underappreciated in the human germline.

Recent studies have begun to profile SV at sequence resolution in healthy human populations, such as the 1000 Genomes Project and the Genome of the Netherlands Consortium [1, 24], though most population-scale studies to date have not deeply characterized balanced SVs or cxSVs. Indeed, while somatic cxSV has been an emphasis in analyses of tumor genomes [25–27], investigations of SV in germline disease have predominantly been restricted to gross chromosomal abnormalities and large, *de novo* CNVs [9, 28–36]. Several studies of germline SV have demonstrated that a subset of SV represents an important class of penetrant, pathogenic loss-of-function (LoF) mutations that are not broadly ascertained in human disease studies [4, 5, 37–39]. By example, imputed genotypes of polymorphic SVs at the major histocompatibility complex (MHC) and haptoglobin (*HP*) loci in large populations have demonstrated disease relevance for schizophrenia and untoward cardiovascular lipid phenotypes, respectively [40, 41]. To date, no population-scale disease studies have evaluated the full mutational spectrum of large SV—specifically including balanced SV and cxSV—though there is a pressing need for such SV maps with the upcoming emergence of large-scale whole-genome sequencing (WGS) studies to characterize the genetic architecture of human disease.

Here, we performed long-insert whole-genome sequencing (liWGS) on 689 participants diagnosed with autism spectrum disorder (ASD) or other developmental disorders to benchmark the population-level landscape of complex and large SVs in a relevant disease cohort. liWGS is optimized to provide deep physical coverage (mean 105X) by large fragments (mean 3.5 kb) capable of detecting large SVs, including some variants that may be intractable to standard short-insert WGS (siWGS) due to repetitive sequences and microhomology that often mediate SV breakpoints, with the primary limitation being its comparatively limited effective resolution (~5 kb) [42, 43]. These data yielded a catalog of seven major SV classes and further revealed 16 recurrent subclasses of cxSV, most of which had not been classified in human disease studies. Further analyses identified a surprising abundance and diversity of inversion variation and derived a broad spectrum of rare cxSV in every genome surveyed, which collectively displayed many of the hallmarks of deleterious biological significance and evolutionary selection. This study also detected three cases of extreme germline chromoanagenesis, which were integrated into an analysis of all previously reported cases of chromoanagenesis in the literature to define the properties of germline chromoanagenesis. These data provided an initial atlas of SV in the morbid germline that can be used as a benchmarking resource for future investigations and suggest that balanced SV and cxSV are relatively common in the human genome, warranting consideration in genetic studies of disease.

## Results

### Sample selection and genome sequencing

**Fig. 1:**
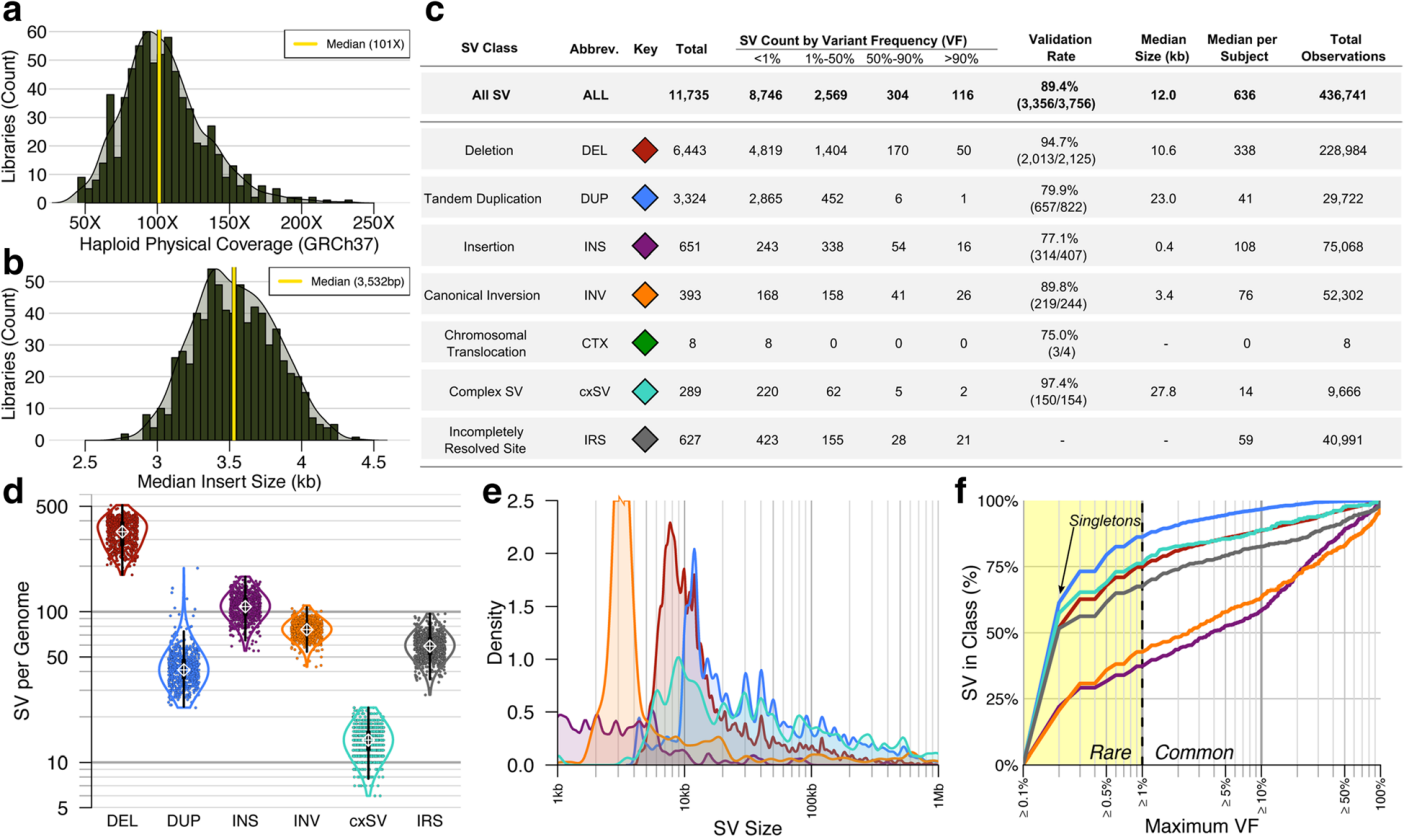
The diverse landscape of SV in participants with ASD and other developmental disorders. We sequenced the genomes of 689 participants with ASD and other developmental disorders. **a** Physical coverage and (**b**) median insert size of liWGS libraries. **c** Count and distributions of large SV detected by liWGS (Additional file 1). **d** Distribution of SVs per participant by SV class. **e** Density plots of SV sizes by class. Characteristic *Alu* and L1 peaks are absent due to the resolution of liWGS (> ~ 5 kb) being larger than most mobile element insertions. **f** Cumulative distributions of SV frequencies by class. Singletons (single observation among all 686 samples) are marked with an arrow. Rare SVs are defined as those with variant frequency (VF) < 1%

We selected 686 participants diagnosed with idiopathic ASD from the Simons Simplex Collection (SSC) [44]. All participants from the SSC met standardized diagnostic criteria for ASD and many included co-morbid diagnoses of intellectual disability, developmental delay, or seizures. All participants had two unaffected parents and at least one unaffected sibling available from the SSC. Independently, we recruited three unrelated participants presenting with neurodevelopmental disorders (NDD) or congenital anomalies and a *de novo* translocational insertion ascertained by clinical karyotyping that appeared to harbor additional complexity. We performed liWGS on all 689 participants to a mean insert size of 3.5 kb and a mean physical coverage of 105X as shown in Fig. 1a and b [42, 43].

### Discovery and validation of a diverse spectrum of SV in the morbid human genome

Among the initial 686 SSC participants, analyses revealed a highly heterogeneous landscape of 11,735 distinct SVs at the resolution of liWGS, representing a total of 436,741 SV observations or a mean of 637 large SVs per genome (Additional file 1 and Fig. 1c and d). Extensive validation was performed to evaluate the SV detection methods used: one-third of all fully resolved SVs (33.8%; 3756/11,108) were assessed using a combination of five orthogonal approaches, as detailed in Additional file 2: Supplemental Results 1 and Supplemental Table 1. These experiments estimated a global false discovery rate (FDR) of 10.6% and false negative rate (FNR) of 5.9% for SV discovery from liWGS. Performance was best for cxSVs (2.6% FDR; see Additional file 2: Supplemental Note 1) and canonical deletions (5.3% FDR), which collectively comprised the majority (57.4%) of all SVs. As anticipated, validation rates were lowest for insertions (22.9% FDR), the majority of which are known to be smaller than the resolution of liWGS (e.g. SVA and *Alu* mobile element insertions) [1, 7, 45] and represent a major challenge for liWGS detection. Excluding this category of variation, the overall FDR improved to 9.1%. Importantly, 16.8% (1968/11,735) of all SVs were either balanced or complex, emphasizing that an appreciable fraction of large SV per genome is overlooked when restricting analyses to canonical CNVs alone. These analyses also found that 10.9% (75/686) of all participants harbored at least one very large, rare SV (≥1 Mb; variant frequency (VF) < 1%), implicating rare SV as a frequent source of large structural divergence between individual genomes (Fig. 1e and f).

### Novel SV sites and rearrangement complexity

This SV map was compared with six recent WGS SV studies outside of the SSC [1, 5, 7, 46–48], the Database of Genomic Variants (DGV) [49], and the InvFEST inversion database [50], which determined that 38.1% (4233/11,108) of all SVs detected in this study (excluding incompletely resolved sites, n = 627/11,735) had not been previously reported. This was particularly true for cxSVs, nearly all which were novel to this study (93.8%; 271/289), including 50.2% for which at least one breakpoint had been observed previously but likely misclassified as canonical SVs (e.g. Additional file 2: Figure S1). Notably, 97.4% of cxSVs were validated in the present study; however, due to the limited resolution of liWGS we predict that this is likely to be an underestimate of the complexity associated with these variants and their overall structure as liWGS is blind to micro-complexity at SV breakpoints, and the resolution to delineate components of cxSVs comprised of small variants (< 5 kb) is limited (Additional file 2: Supplemental Note 1) [1, 10, 51, 52]. In sum, these data revealed that large cxSVs in humans are substantially more abundant and diverse than has been previously appreciated.

### Defining and contrasting 16 distinct subclasses of large, recurrent cxSV

**Fig. 2:**
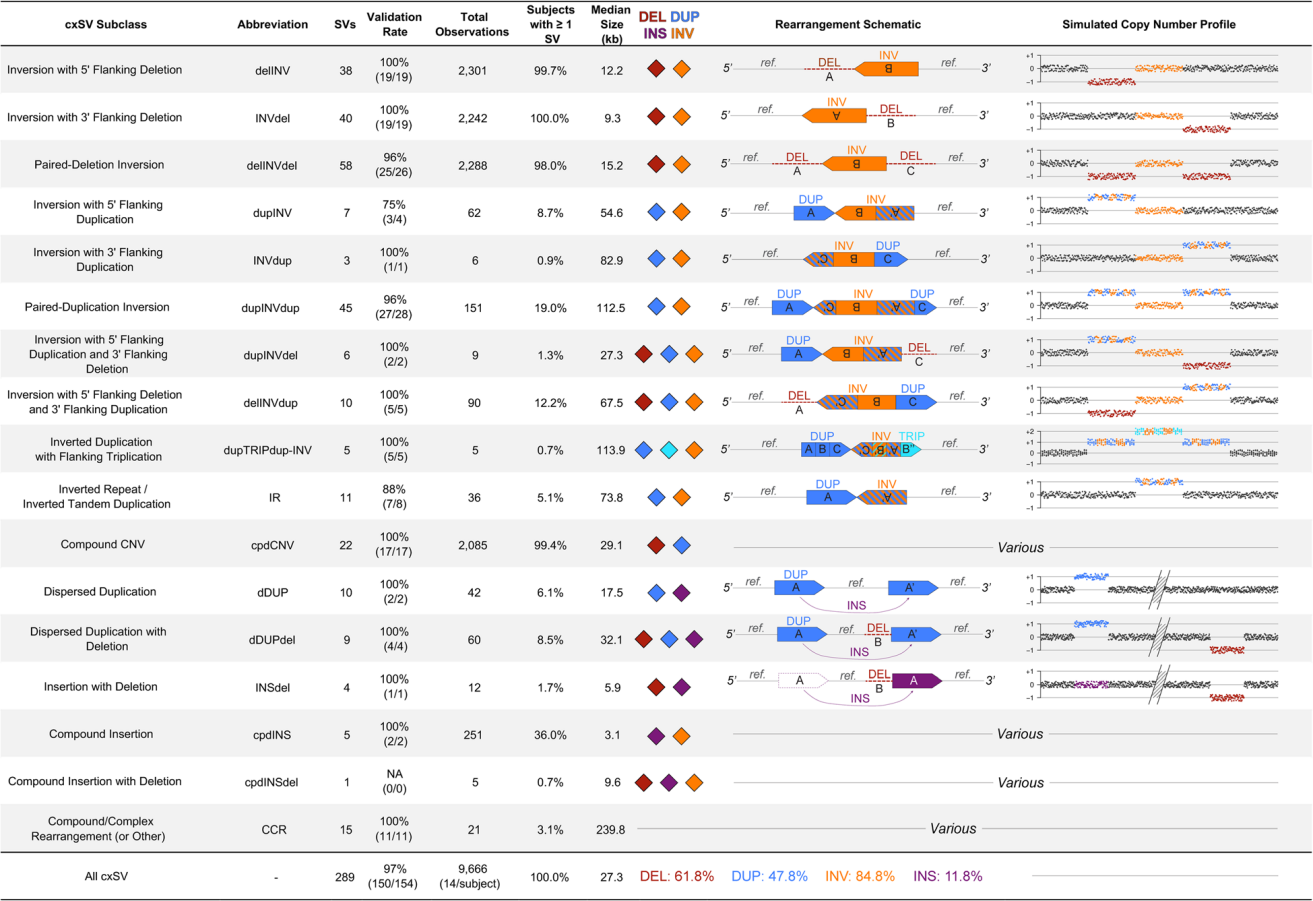
Classifying 16 recurrent subclasses of large, complex SVs in the human genome. At liWGS resolution, we identified 16 recurrent classes of cxSV, defined here as non-canonical rearrangements involving two or more distinct SV signatures or at least three linked breakpoints. We validated 97.4% (150/154) of all cxSV sites assessed by at least one assay. Each participant harbored a median of 14 cxSVs at liWGS resolution (range: 6–23 cxSVs per participant). We identified 289 distinct cxSVs across 686 participants, totaling 9666 cxSV observations. Each row represents a subclass of cxSV, with columns representing the subclass abbreviation, number of distinct variants discovered, validation rate, total number of observed variants across all participants, the percentage of participants that were found to harbor at least one such variant in their genome, the median size of all variants in that subclass, each subcomponent SV signature that comprises the class, a linear schematic of each class of cxSV, and a simulated example of the copy-number profile as would be observed by chromosomal microarray or WGS

The frequency of novel, large cxSVs in this cohort led us to further characterize their mutational spectra. We observed that 42.6% (123/289) of all cxSVs were polymorphic (i.e., appearing in at least two participants), and each participant harbored a median of 14 large cxSVs (range: 6–23 cxSVs per genome), establishing that cxSV is a standing class of variation present in most, if not all, human genomes. We classified 16 unique subclasses of recurrent and relatively common cxSVs for consideration in future genomic studies, as presented in Fig. 2. Each cxSV subclass appeared in at least five participants and featured a signature variant allele structure. The majority of these subclasses (10/16) were unbalanced inversions and thus most cxSVs (84.8%) involved at least one inverted segment. Correspondingly, CNV-flanked inversions comprised the largest group of cxSVs (77.2%), with complex duplications being larger and rarer on average than complex deletions (Additional file 2: Figure S2). Both deletions and duplications flanking complex inversions were equally likely to arise at either inversion breakpoint, consistent with either replicative repair-based mechanisms such as MMBIR/FoSTeS [6, 39, 53] or synchronous repair of multiple simultaneous double-strand breaks [18, 54]. Most cxSVs were intrachromosomal, with relatively few rearrangements (3.1%; 9/289) involving two or more chromosomes. As discussed above, these 16 cxSV subclasses certainly represent a conservative initial catalog of the full complement of cxSV in humans given the resolution of liWGS.

### Abundance of canonical and complex inversion variation

Routine detection of large inversion variation has historically been a challenge for high-throughput technologies, including siWGS [1, 50, 55–57]. Although recent advances in long-read and strand-specific WGS represent promising novel platforms for inversion discovery [7, 58, 59], liWGS remains particularly well suited for inversion detection as the distance spanned between paired reads (~3.5 kb) avoids most confounding repetitive sequences and imbalances that frequently occur at inversion breakpoints [6, 10]. In this cohort, liWGS identified a median of 87 inversion variants per participant, a surprising fraction of which (12.6%; 11/87) were complex (Additional file 2: Figure S3A). These complex inversions were larger on average than canonical inversions (Additional file 2: Figure S3B) and were also significantly enriched in rare variants (VF < 1%): 75.9% of complex inversions were rare (186 rare/245 total), while only 43% of canonical inversions were rare (169 rare/393 total) (*p* = 1.2 × 10^–16^), which suggests that complex inversions might be under relatively increased purifying selection. It is possible that this trend may also be attributable in part to a correlation between SV frequency and average size [1], as larger inversions might be less viable in the germline either due to increased deleterious consequences or by obstructing recombination [60]. The number of inversions per genome identified in this study was approximately twofold greater than estimates from the 1000 Genomes Project from low-depth siWGS on 2504 samples [1]. Given the validation rate for inversions (canonical inversion: 89.8%; complex inversion: 96.9%), we hypothesized that this difference may be due to inversion breakpoints being enriched near longer repetitive sequences, which might confound siWGS but would still be accessible to liWGS. Indeed, we found that 87.6% of all inversion-associated variants (both complex and canonical; n = 636) had one or both breakpoints within ±500 bp (*i.e*. conservative liWGS breakpoint resolution) of a relatively long (≥300bp) annotated repetitive sequence  [61], and both breakpoints were in proximity to long repetitive sequence for 54.9% of inversions. Both observations significantly deviated from the null distribution from 1 million matched simulations (*p* < 1.0 × 10^–6^), as shown in Additional file 2: Figure S3C. This included inversion breakpoints in segmental duplications, despite the limited power of short-read sequencing to detect variation at these loci, consistent with previously proposed mechanistic hypotheses of inversion formation [58, 59, 62]. Collectively, the patterns of canonical and complex inversions observed herein suggest that a substantial fraction of such variation may be preferentially accessible to sequencing technologies like liWGS that provide long-range information on genome structure.

### Resolving intractable rare cxSV with linked-read WGS

**Fig. 3:**
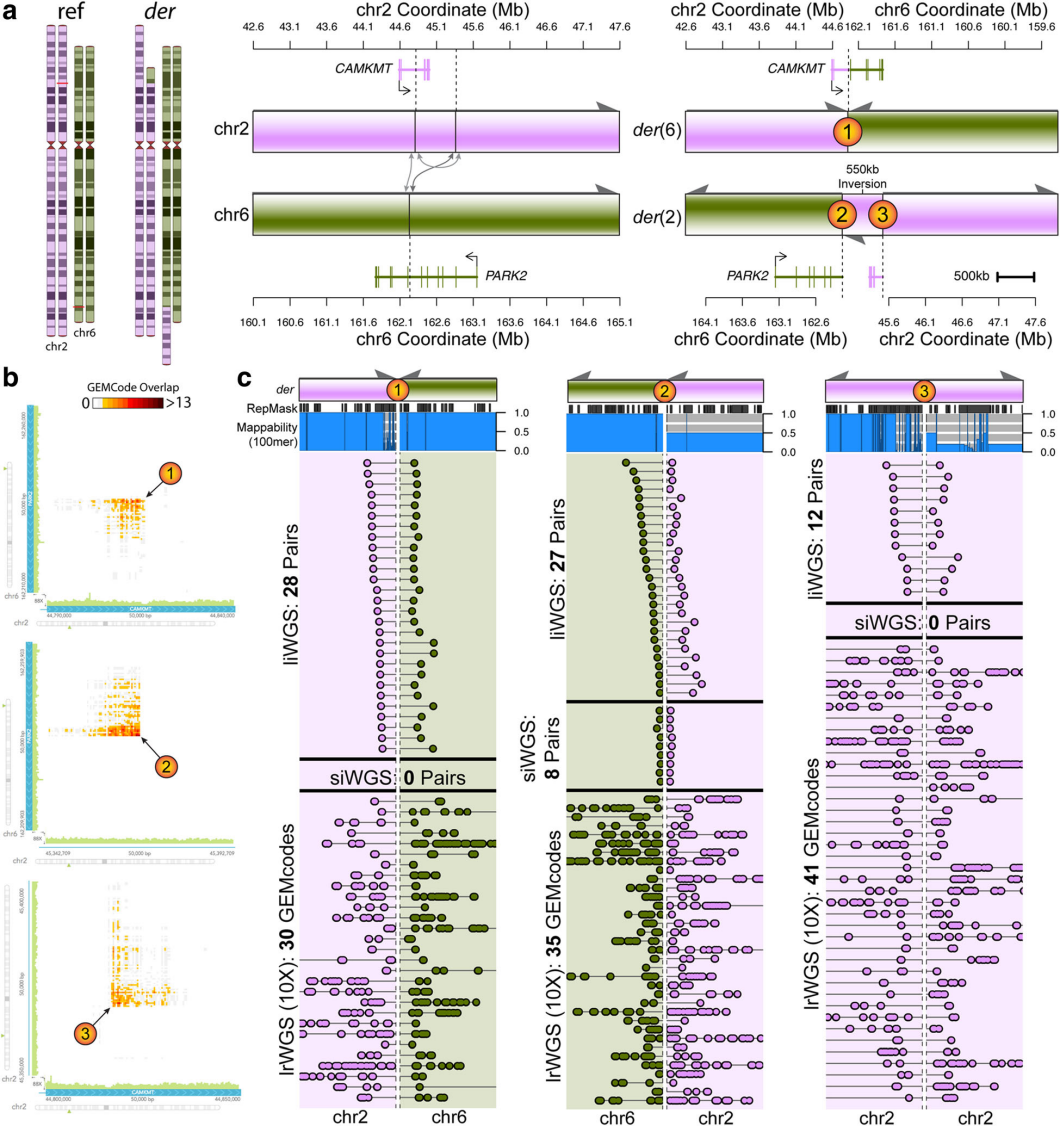
liWGS and lrWGS resolved a *de novo* gene-disrupting cxSV that was cryptic to standard siWGS. We performed lrWGS from 10X Genomics (Pleasanton, CA, USA) as a method of orthogonal validation for three large complex SVs detected by liWGS, two of which failed to fully validate by traditional methods. One notable example is shown here; the other two are provided in Additional file 2: Figures S4 and S5. **a** A *de novo* complex reciprocal translocation with three breakpoints between chromosomes 2 (*pink*) and 6 (*green*) was discovered by liWGS in a participant with ASD and predicted to result in LoF of *PARK2* and *CAMKMT*. However, two of three breakpoints (breakpoints #1 and #3; *orange*) were not detectable by siWGS. **b** lrWGS heatmaps from Loupe software [113] analysis of lrWGS data showed clear evidence for each of the three SV breakpoints. **c** lrWGS resolved and phased all three breakpoints, including both breakpoints that failed molecular validation due to low-complexity repetitive sequence (*blue*), which were resolved by spanning the low-complexity sequence with 28 liWGS reads and 30 lrWGS molecules at breakpoint #1 and 12 liWGS reads and 41 lrWGS molecules at breakpoint #3

We performed linked-read WGS (lrWGS) from 10X Genomics [63] to resolve large, rare cxSVs detected by liWGS in three participants for which the liWGS delineated rearrangements that were not fully resolved by orthogonal validation. We sequenced these three participants and two parents to a median of 31.1X nucleotide coverage. From these data, we resolved all breakpoints of each predicted large cxSV, notably including a *de novo* complex translocation in a participant with ASD that involved 550 kb of inverted sequence and three breakpoints predicted by liWGS, two of which could not be validated by traditional approaches (polymerase chain reaction (PCR) and Sanger) or by siWGS due to low sequence uniqueness flanking the junctions (Fig. 3). All three breakpoints were confirmed and phased by 104 independent lrWGS molecules, revealing disruption of the genes *PARK2* and *CAMKMT*. The other two large cxSVs validated by lrWGS are provided in Additional file 2: Figures S4 and S5. Building upon our earlier observations of inversion variation, these data further suggest that technologies that provide long-range structural information will be of value for resolving large complex chromosomal abnormalities, and comprehensive analyses are required in larger samples to determine the improved yield of SVs from lrWGS as compared to siWGS, liWGS, or other emerging technologies.

### Rare SVs exhibit multiple hallmarks of deleterious biological consequences

**Fig. 4:**
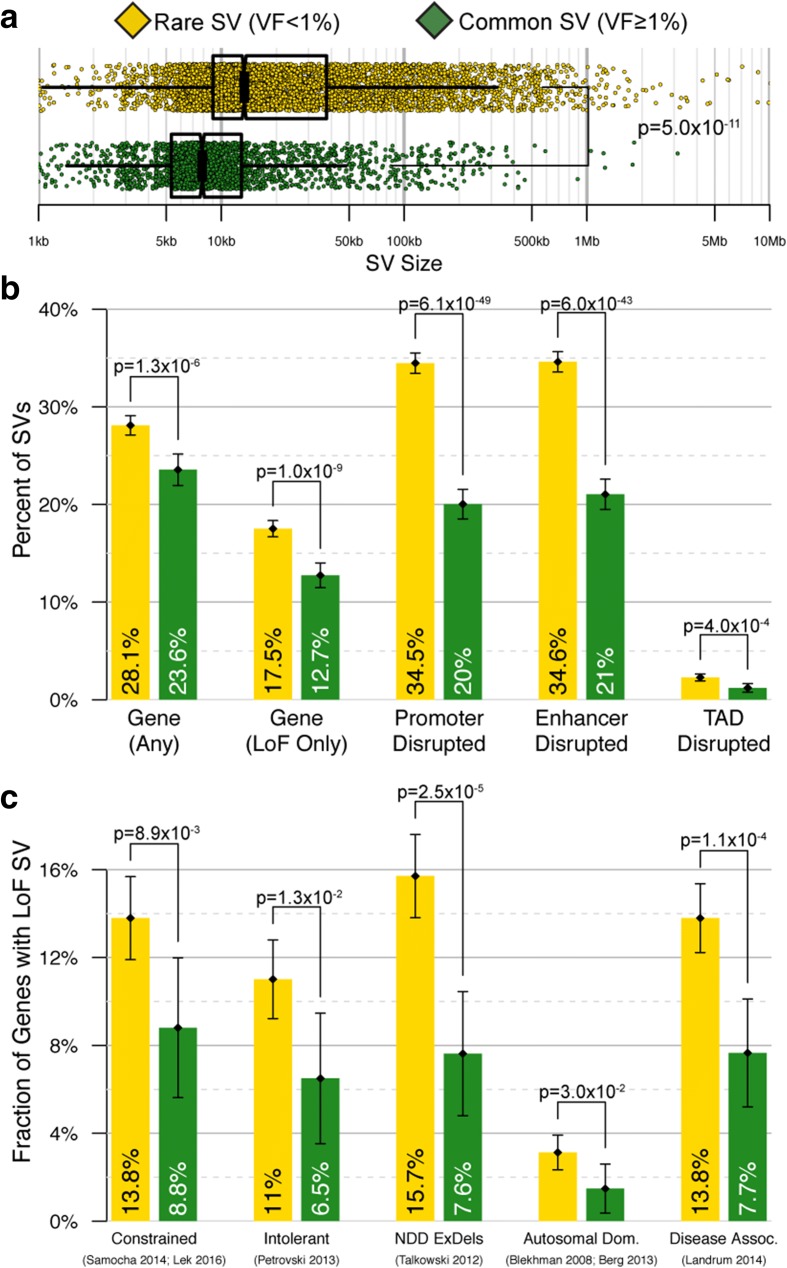
Rare SVs are enriched for hallmarks of deleterious biological outcomes. Comparing all rare (VF < 1%) and common (VF > 1%) SVs discovered in this cohort revealed differences in their respective functional annotations (Additional file 2: Table S2). **a** Rare SVs were larger on average than common SVs [1]. **b** Rare SVs were more likely than common SVs to disrupt genes, particularly when the disruption was predicted to result in LoF. Rare SVs were also more likely than common SVs to result in disruption of promoters [112, 114], enhancers [112, 114], and TAD boundaries [110]. **c** Genes predicted to harbor at least one LoF mutation due to a rare SV were enriched in many subcategories when compared to common SV, including genes predicted to be constrained against truncating mutations in healthy individuals (Constrained) [65, 66], genes predicted to be intolerant of functional variation in healthy individuals (Intolerant) [67], genes with significant burdens of exonic deletions in NDD cases versus healthy controls (NDD ExDels) [38], genes associated with an autosomal dominant disorder (Autosomal Dom.) [68, 69], and genes with at least one pathogenic variant reported in ClinVar (Disease Assoc.) [70] (Additional file 2: Table S3)

Consistent with trends observed among rare coding point mutations [64–67], rare SVs (VF < 1%) appeared to be considerably more deleterious than common polymorphic SVs (VF > 1%) based on computational annotations (Additional file 2: Supplemental Results 2). Rare SVs in this cohort were larger than common SV, in line with observations from the 1000 Genomes Project [1], and were also nearly twice as likely to disrupt multiple classes of regulatory non-coding elements, and 1.5-fold more likely to result in predicted LoF of genes (all comparisons were significant and test statistics are provided in Fig. 4a and b and Additional file 2: Table S2). The set of genes truncated by rare LoF SVs in this study was also approximately twofold enriched in disease-associated genes [68–70], genes intolerant to functional mutation [65–67], and genes with burdens of exonic deletions in NDDs [38] (Fig. 4c and Additional file 2: Table S3.) These findings were concordant with the hypothesis that loci sensitive to disruptive point mutations in healthy individuals would also show selective pressure against deleterious SV. Finally, we identified ten specific loci that were significantly enriched for rare SVs beyond genome-wide expectations (Additional file 2: Supplemental Results 3, Figure S6 and Tables S4–5), five of which involved genes with evidence for roles in a broad spectrum of neurological disorders (*PARK2*, *IMMP2L*, *CTNNA3*, *CYFIP1*, *PTPRT*) [32, 71–75]. Additional SV studies in larger matched case-control cohorts will be required to elucidate any role of SV at these loci in disease risk, and such studies are ongoing.

### Extreme chromoanagenesis in aberrant human development

**Fig. 5:**
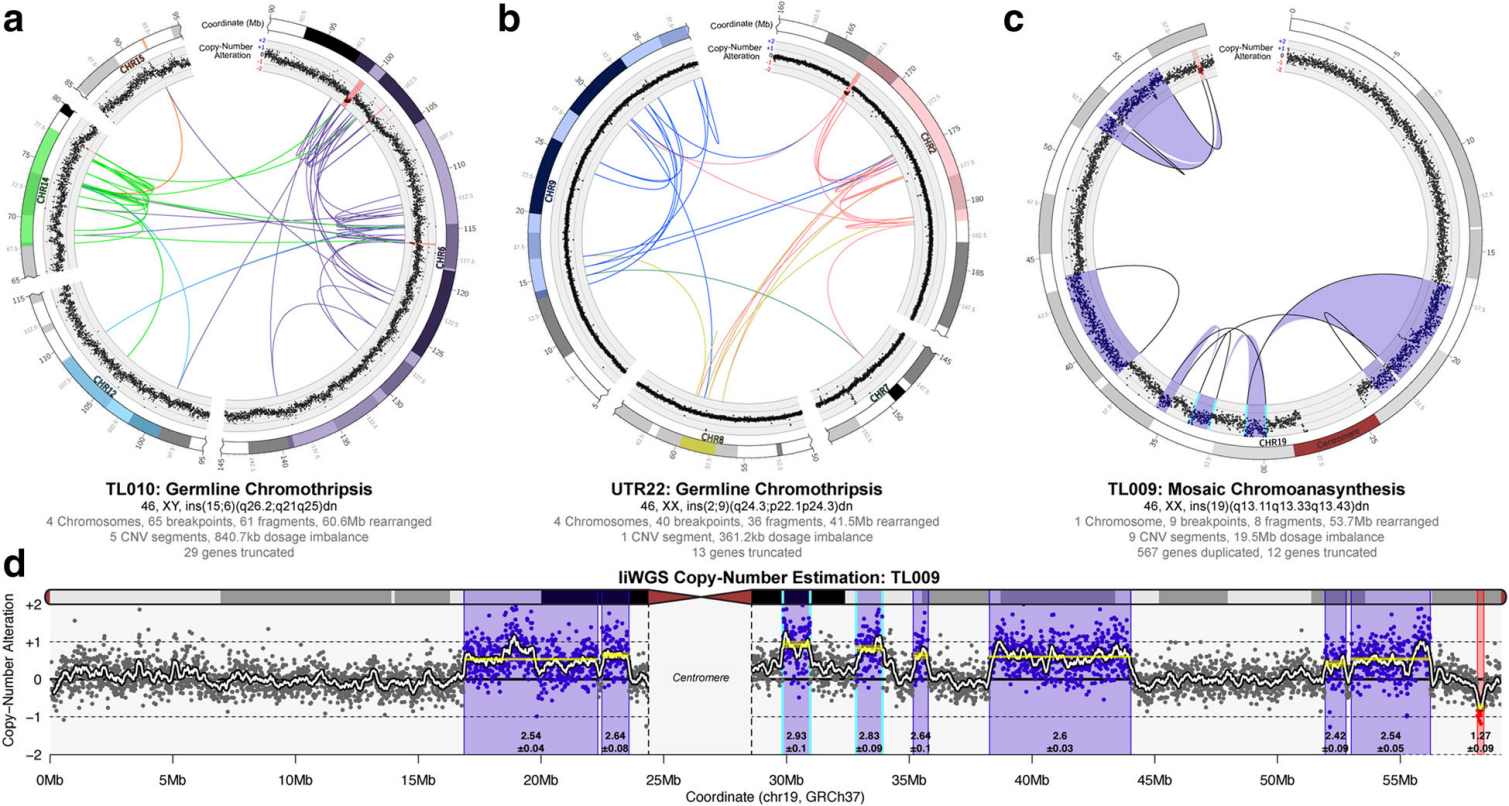
Extreme chromoanagenesis manifests by multiple mutational mechanisms in three participants with developmental anomalies. We applied WGS to resolve microscopically visible cxSVs in three unrelated participants with developmental abnormalities. **a**, **b** Circos representations of two cases of extreme and largely balanced chromothripsis, involving > 40 breakpoints, > 40 Mb, and > 12 genes across four chromosomes [9, 115]. Points plotted around the inner ring represented estimated copy number alterations; deletions are highlighted in *red*. Links represent non-reference junctions on derivative chromosomes. **c** Circos representation of a somatic mosaic chromoanasynthesis event of chromosome 19 [115]. Duplications are shaded in *blue* and interspersed duplications are designated by shaded ribbons leading from the duplicated sequence to their insertion site. **d** CMA and WGS analysis of the mosaic chromoanasynthesis from panel **c** ﻿(participant TL009﻿) revealed all nine CNVs involved in the rearrangement to have arisen on the maternal homologue and that 6/8 duplications were apparently mosaic (2.57 ± 0.02 copies, 95% CI; median coverage shown in *yellow*; *yellow shading* indicates 95% CI). Surprisingly, 2/8 duplications (outlined in *teal*) exhibited significantly greater copy numbers than the other six (*p* = 9.18 × 10^–8^), were linked by an underlying interstitial inversion and appeared to represent approximately three copies, suggesting this rearrangement might have originated as a *de novo* dupINVdup cxSV in the maternal germline (Additional file 2: Figure S7)

The most catastrophic SVs catalogued to date involve the cxSV subclass known as chromoanagenesis. To summarize existing knowledge of chromoanagenesis and contextualize the findings from this study, we conducted a literature review of published reports of germline chromoanagenesis at sequence resolution, almost all of which arose *de novo* in affected individuals. The results of this review are consolidated in Table 1 and Additional file 2: Table S6 [9, 10, 13, 17–23, 76–78]. Based on this knowledge, and separate from the genome-wide SV analysis of the 686 SSC participants described above, we performed liWGS on an additional three unrelated participants (participants TL010, UTR22, and TL009) with developmental anomalies and large *de novo* translocational insertions identified by clinical karyotyping, which we suspected may represent more complex rearrangements. The rearrangement in subject UTR22 has since been recently described [9]. Sequencing analysis revealed that the first two participants, TL010 and UTR22, harbored extreme yet almost entirely balanced germline chromothripsis events, each involving > 40 breakpoints, >40 Mb of rearranged sequence, four chromosomes, and LoF of > 12 genes, yet < 1 Mb of total dosage imbalance (Fig. 5a and b, Additional file 2: Table S7, and Additional file 3).

**Table 1:** Characteristics of chromoanagenesis classes

	*Chromothripsis*	*Chromoanasynthesis*	*Chromoplexy*
Mutational event	Single	Single or multiple	Single or multiple
Chromosomes	Few (1–4)	Few (usually 1)	Many (usually ≥ 4)
Breakpoints	Many (≥5; sometimes > 25)	Fewer (usually 5–25)	Fewer (usually 5–25)
Breakpoint distribution	Clustered	Clustered	Interspersed (usually in active chromatin)
Breakpoint signature	Blunt ends	Microhomology	Blunt ends
Dosage alteration	*Cancer*: often unbalanced (deletion bridges); *Germline*: mainly balanced (<5% of total rearrangement)	Unbalanced (predominantly copy gain)	Mainly balanced (occasional deletion bridges)
Proposed mechanism	Micronucleus missegregation + chromosome pulverization + NHEJ	Micronucleus missegregation + chromosome pulverization + MMBIR/FoSTeS	Multiple DSBs during active transcription + NHEJ
Proposed parent-of-origin bias	Paternal	None	None
Proposed transmission bias	Maternal	None	None
Germline reports	43	10	6
Case:Control	39:4	10:0	6:0
References	[9, 10, 17, 18, 23, 76–78]	[19–21]	[9, 22, 23]

In contrast to the first two participants, TL009 harbored a somatic mosaic unbalanced chromoanasynthesis of chromosome 19, involving 19.1 Mb of duplicated DNA, copy gain (CG) of 567 genes, 361.2 kb of deleted DNA, and LoF of 12 additional genes (Fig. 5c and Additional file 3). Intriguingly, while all eight duplicated loci arose on the maternal homologue, 6/8 of these duplications were predicted to be mosaic from liWGS (2.57 ± 0.02 copies, 95% confidence interval (CI)), yet the other 2/8 duplications appeared at nearly three full copies (2.93 ± 0.10 and 2.83 ± 0.09 copies, 95% CIs), which may contrast previous assumptions that chromoanasynthesis arises in a single mutational process. Both of the apparently higher-copy-state loci were significantly greater in copy number than the six mosaic duplications (*p* = 3.60 × 10^–12^ and *p* = 9.18 × 10^–8^) but not different from each other (*p* = 1.04 × 10^–1^) (Fig. 5d). Remarkably, these two duplications were connected by a 5.1 Mb interstitial inversion, resulting in a mutational signature that matches the dupINVdup cxSV subclass previously described (Fig. 2) [4]. We speculated that the rearrangement in TL009 may have arisen initially as a *de novo* dupINVdup either in the maternal germline or very early in embryonic development, and was subsequently compounded by a second mutational event, possibly through mitotic missegregation driven by genome instability from the large dupINVdup near the centromere (Additional file 2: Figure S7). These three cases further illustrate that extreme chromothripsis can arise in the germline while often resulting in near dosage-neutral derivatives and that unbalanced chromoanasynthesis can arise in soma, perhaps in a temporally punctuated series of rearrangements more closely resembling the compounded mutations of chromoplexy than a single catastrophic mutational process [14, 79].

## Discussion

By applying an approach optimized for genome-wide SV discovery to a cohort of nearly 700 participants with ASD and related developmental disorders, these data provided a glimpse of the diverse mutational landscape of large SVs in the morbid human germline. Analyses revealed a substantial number of novel canonical and complex SV sites, and a wide breadth of large cxSV mutational signatures. Ascertaining SVs with liWGS also uncovered a surprising abundance of canonical and complex inversion variation, some of which were likely to be intractable to siWGS due to local sequence characteristics in proximity to the breakpoints. Importantly, owing to the limited resolution of liWGS, the barriers to SV detection using short-read sequencing, and the limitations of reference-based alignments more broadly [24], the diversity of cxSVs described here still likely accounts for only a fraction of the mutational landscape of cxSV in the human germline, and likely underestimates the sequence-level complexity of the variants reported herein. We anticipate many additional subclasses will continue to be discovered from larger population-scale studies and higher resolution technologies. Finally, annotation of the balanced SVs and cxSVs identified in this cohort demonstrated that these classes of variation contributed a m﻿odest but meaningful number of perturbations of coding and noncoding regulatory loci per genome, the effects of which were predicted to be particularly deleterious among rare variants, suggesting that routine characterization of the complete spe﻿ctrum of SV in genetic studies of human disease may improve power to resolve the genetic etiologies of some disorders. In sum, these data thus represent a benchmark for major classes of large SVs that will be expanded by future efforts.

These analyses indicate that large and complex chromosomal abnormalities are relatively common in the human germline, and that numerous large cxSVs likely exist in every human genome, with the most extreme cxSVs (*e.g*. chromoanagenesis) representing one tail of the distribution of SV complexity and size. While still rare, our data confirm that non-tumorigenic chromoanagenesis exists as both constitutional and somatic variation and that cytogenetically detected *de novo* interchromosomal insertions may hallmark such extreme rearrangements, though larger collections of samples are warranted to further investigate this phenomenon. The review of chromoanagenesis literature performed herein [10, 13, 17–23, 76–78] (Table 1 and Additional file 2: Table S6) supports three conclusions: (1) constitutional chromoanagenesis is frequently balanced, possibly due to embryonic selection against loss of genes intolerant to haploinsufficiency [79–81]; (2) extreme genomic rearrangements can be tolerated in the developing germline [77, 78], although cases of unbalanced extreme chromoanagenesis have mostly been reported in cancer; and (3) at least 2/55 of these rearrangements appeared to be the product of multiple compounding mutational events [23] and another 4/55 rearrangements were observed to acquire additional rearrangements *de novo* upon unstable transmission from parent to child [23, 77], suggesting it is unlikely that such catastrophic rearrangements always arise in a single mutational event. This latter conclusion draws a key parallel between the two prevailing proposed mechanisms of cancer chromoanagenesis, wherein some rearrangements likely arise from DNA shattering in missegregated micronuclei during mitosis [12, 54, 82–85], yet others acquire additional breakpoints over punctuated tumor evolution [14, 79, 86], not unlike the six constitutional rearrangements with some degree of evidence against a singular mutational event [23, 77].The mosaic chromoanasynthesis characterized in this study may be an exemplar of such mutational progression, as two of the largest duplications appeared to represent germline duplications (copy state ~ 3), whereas the remaining rearrangements were present at lower mosaic fractions (copy state ~ 2.5), possibly indicating progressive mutational acquisition. Further study into the mechanisms of such alterations, and comparisons to the micronuclei hypothesis, would be of great interest in our evolving understanding of this phenomenon.

## Conclusions

This study provides new insights into the extensive and diverse subclasses of SVs in the morbid human genome and illuminates that inversion variation is substantially more complex than has been appreciated from other technologies. The patterns of variation defined here extend previous maps of SVs in the general population [1, 24], and functional annotations of the SVs in this cohort demonstrate that rare SVs are more likely than common SV to disrupt both coding and regulatory non-coding elements. These analyses further suggest that genes truncated by rare SV are more likely to be constrained against inactivating point mutations in healthy individuals and associated with disease phenotypes in large clinical databases. The presentation of three cases of chromoanagenesis further support earlier evidence that extremely complex balanced rearrangements are tolerated in the human germline, and suggest that some catastrophic constitutional rearrangements may arise through multiple mutational events. This study emphasizes the need for detailed characterizations of SVs to aid in interpretation of the morbid human genome, and these data provide a reference map of inversions and cxSVs to be built upon by population-scale sequencing studies.

## Methods

### Sample selection and phenotyping

Samples included in genome-wide analyses (n = 686) were acquired from the SSC, a cohort of 2591 simplex autism families, each with one affected child, one or more unaffected siblings, and two unaffected parents collected from 12 sites across the United States [44]. We randomly selected 230 unrelated SSC probands, and selected the remaining 456 on the basis of no known pathogenic *de novo* gene-truncating point mutation or large *de novo* CNV from prior whole exome sequencing (WES) and CMA analyses [36]. All probands selected from the SSC met standardized diagnostic criteria between the ages of four and 16 years for ASD and often one or more additional neurodevelopmental anomalies, which in this study included developmental delay (60.7%), intellectual disability (31.6%), and seizures (12.3%). Phenotype information for each sample was previously ascertained by the SSC investigators (see “Acknowledgements”) and we obtained these data with permission through the online SFARIbase portal (http://sfari.org/resources/sfari-base). DNA was obtained through SFARI from the Coriell Cell Repository at Rutgers University (Camden, NJ, USA). The three cases with cytogenetically detected *de novo* translocational insertions were referred by the University of Torino (Italy), the Columbia University Medical Center (USA), and the UCLA Clinical Genomics Center (USA) based on cytogenetic findings from G-banded karyotyping. Informed consent was obtained for all patients (either during collection by the SSC or at the referring sites) and all samples (except UTR22) were sequenced with approval from the Partners Healthcare Institutional Review Board. Ethical approval for sequence analysis of case UTR22 was given by the ethical committee of the San Luigi Gonzaga University Hospital-Orbassano (TO) Italy.

### liWGS library preparation and sequencing

Custom liWGS libraries were constructed using our previously published protocols for all samples except case UTR22, the protocol for which is described below [42, 43]. One library was prepared and sequenced per participant, and in a subset of 22 participants, we prepared two separate libraries as technical replicates to evaluate the replicability of our computational methods. This resulted in a total of 711 libraries included in this study. Libraries were quantified by the PicoGreen assay and sequenced on either an Illumina HiSeq 2000 or 2500 platform with 25 bp paired-end chemistry at the Broad Institute (Cambridge, MA) or the Massachusetts General Hospital (MGH). Library barcodes were demultiplexed per Illumina’s stated best practices. Reads failing Illumina vendor filters were excluded. Read quality was assessed with FastQC v0.11.2 (http://www.bioinformatics.babraham.ac.uk). Reads were aligned to human reference genome assembly GRCh37 (GCA_000001405.11) (http://apr2013.archive.ensembl.org/Homo_sapiens) with BWA-backtrack v0.7.10-r789 [87]. Duplicates were marked with SAMBLASTER v0.1.1 [88]. All alignment manipulation, including sorting and indexing, was performed with sambamba v0.4.6 [89]. Alignment quality was assessed using PicardTools v1.115 (http://broadinstitute.github.io/picard/), Samtools v1.0, and BamTools v2.2.2 [90, 91]. All libraries were evaluated for sequencing and alignment quality on numerous metrics, including mapped read pairs, per-read and pairwise alignment rate, chimeric pair fraction, haploid physical coverage, per-read and pairwise duplicate rate, median insert size, and insert size median absolute deviation (MAD). All libraries except for those generated from the three referred clinical cases with large cytogenetic abnormalities were analyzed genome-wide for the full mutational spectrum of SV, the methods for which are described below.

Case UTR22 was recently described in a separate study [9], but the sequencing protocols used for this case are briefly restated here as follows: a liWGS library was prepared using the Illumina mate-pair library kit. The library was sequenced on an Illumina NextSeq using paired 75 bp reads. The same DNA sample was also sequenced by paired-end siWGS on an Illumina HiSeq X instrument (paired 151 bp reads). Reads were aligned to the reference genome assembly GRCh37 using BWA-0.7.5a [87]. SV discovery in the UTR22 siWGS library was conducted using Manta with standard settings for siWGS [92] and an independent custom pipeline for liWGS [17].

### lrWGS library preparation and sequencing

Prior to 10X Genomics lrWGS library construction, genomic DNA samples were checked for fragment size distribution and were quantified. Genomic DNA fragment size distributions were determined with a Caliper Lab Chip GX (Perkin Elmer) to quantify DNA above 40 kb in length. Size selection was performed on 1.2 ug of genomic DNA with an 0.75% Agarose cassette on the Blue Pippin platform (Sage Science) with target specifications set to start at 40 kb and end at 80 kb. Samples were quantified using the Quant-it Picogreen assay kit (Thermo Fisher) on a Qubit 2.0 Fluorometer (Thermo Fisher) and normalized to a starting concentration of 1 ng/uL with TE (0.1 mM EDTA). Starting concentrations of 1 ng/uL were confirmed by picogreen and libraries were subsequently created in accordance with the 10X WGX protocol (10X Genomics). Library size was determined using the DNA 1000 Kit and 2100 BioAnalyzer (Agilent Technologies) and quantified using quantitative PCR (qPCR) (KAPA Library Quantification Kit, Kapa Biosystems). The finished WGX libraries were run on an Illumina HiSeqX platform at paired 151 bp reads with an eight-base single index read at the Broad Institute. Upon completion of sequencing, the resulting BCL files were processed by the Long Ranger Pipeline (10X Genomics) for alignment, variant discovery, and phasing.

### Structural variation discovery from liWGS

A joint-calling consensus framework, *Holmes*, was developed for computational SV discovery optimized for liWGS libraries. This pipeline involves the integration of several SV signals simultaneously in batches of liWGS libraries. The codebase for this pipeline is open-source and publicly available per details listed in “Availability of Data and Materials.” We ran this SV discovery pipeline on sequential batches of 278, 229, and 201 libraries and merged the SV calls from each batch *post hoc*. For all analyses, only the primary GRCh37v71 assembly was considered and the mitochondrial chromosome was also excluded. Although segments of this pipeline have been described in previous publications [4, 5, 10, 37, 38, 43], each stage is enumerated below.

#### Anomalous read-pair clustering algorithm

Non-duplicate pairs of primary alignments were first clustered per library with our previously described single-linkage read-pair clustering algorithms *BAMStat* and *ReadPairCluster* at a minimum cluster size of three pairs and a minimum clustering distance corresponding to the library’s median insert size plus seven MAD [5, 10, 38]. The clustered read pairs were filtered to exclude pairs in which both reads were multiply mapped (BWA MapQ = 0), pairs where one or both reads mapped to annotated somatic hypermutable sites (antibody parts; “abParts”), and pairs where one or both reads mapped to a set of genomic loci known to cause clustering bias in paired-end WGS data adapted from a list compiled by Layer et al. [93]. The remaining anomalous pairs from the initial per-sample clustering were then pooled across all samples and jointly clustered at a minimum cluster size of three pairs and a minimum clustering distance of the maximum clustering distance used for any individual sample in each processed batch. These joint clusters were heuristically classified with a decision tree algorithm that modeled average mapping quality of the component read pairs, ratio of anomalous pairs in the cluster to proper pairs spanning the same interval as the read-pair cluster, ratio of anomalous pair coverage at the putative breakpoint as compared to the median haploid physical coverage of the library, uniqueness of read mapping positions, and maximum span of reads on either side of the putative breakpoint. Thresholds for this decision tree were trained on known valid and invalid breakpoints as determined by previous molecular validation [4, 5]. Each cluster was categorized based on its SV signature: deletion, insertion, inversion, or translocation. These paired-end mapping signatures have been previously described [3, 43, 94]. Hybrid clusters representing two proximal independent variants were separated post hoc via assessment of non-overlapping subgrouping spans between individual samples.

#### Physical sequencing depth algorithm

In parallel with our cluster-based analysis, we also investigated read depth across our cohort using a version of the cn.MOPS algorithm modified to accommodate liWGS data. This modification begins by dividing the genome into 1 kb bins and counts the number of properly aligned read pairs whose insert spans each bin (*i.e*. approximate binned physical coverage), rather than counting the raw number of reads per bin, which is the default setting. cn.MOPS was then run on these 1 kb binned values and further run at larger bin sizes of 3 kb, 10 kb, and 30 kb, which correspond to minimum call sizes of 3 kb, 9 kb, 30 kb, and 90 kb, respectively. The resultant CNV segments were merged across all four bin size runs with BEDTools merge to preserve breakpoint resolution while avoiding overly segmented CNV calls [95]. Supplementing the genome-wide read-depth calling provided by cn.MOPS, we developed a statistical machine-learning framework for local copy state genotyping across all putative CNV intervals based on the same physical depth of coverage matrix used in cn.MOPS CNV discovery. Candidate CNV intervals and their associated sample IDs were input into this genotyping algorithm and a unidirectional t-test was used to evaluate the significance between normalized physical coverage across samples predicted to harbor the CNV and predicted reference samples. The power and permuted *p* value of the t-test were evaluated; we set thresholds of 0.8 and 0.01, respectively, for being sufficiently powered and statistically significant to effectively discriminate alterations in copy state between the two groups of libraries (predicted CNV carriers and predicted diploid/reference samples). For singleton CNVs, as well as sites with insufficient power (<0.8), a single sample z-test was used per individual library and required *p* ≤ 1 × 10^–6^ for a non-reference copy number assignment; this threshold was adjusted to *p* ≤ 1 × 10^–4^ if the diploid cluster standard deviation was particularly noisy (>0.1). Male and female samples were segregated for all depth-based CNV analyses on allosomes.

#### Consensus categorization of canonical CNVs

Canonical CNVs (i.e. CNVs with no additional complexity beyond deletion or tandem duplication) were categorized by a tiered consensus framework to integrate depth-based CNV segments with paired-end clusters (Additional file 2: Figure S8). CNV sites were first nucleated on the presence of paired-end clustering support. Next, all cn.MOPS CNV intervals were merged across all samples simultaneously by clustering 5’ and 3’ breakpoints on proximity independently at a maximum distance of 10 kb per breakpoint between overlapping CNV intervals. The mean breakpoint coordinate was taken when two or more intervals were merged by this approach. These non-redundant cn.MOPS intervals were then overlaid atop paired-end clusters by BEDTools intersect requiring 50% reciprocal overlap and at least one sample shared between both calls, with any cn.MOPS intervals meeting these criteria being merged into the paired-end clusters. In this instance, the union of samples between cn.MOPS and paired-end clustering calls was used and the breakpoint coordinates from the paired-end clusters were retained, since short-read pairwise mappings have finer breakpoint resolution (generally < 1 kb; improves with increased number of observations) than depth-based CNV segmentation (generally ≥ 3 kb) in our approach. When overlap was found between a cn.MOPS interval and a paired-end cluster, the fraction of overlapping samples between these two calls was recorded. Any cn.MOPS interval that did not match a paired-end cluster was treated as an independent CNV interval for the remainder of the consensus CNV pipeline. At this stage, all putative CNVs were copy-state genotyped in all samples as described above, with CNV genotypes being used to affirm or refute a putative CNV call. Finally, all resultant CNV calls were intersected using BEDTools coverage against a blacklist compiled of annotated dispersed multicopy loci (e.g. segmental duplications/low-copy repeats), annotated heterochromatin, known sites of systematic short-read mappability biases [93], and gaps in the reference assembly; any CNV covered ≥ 30% by size by these intervals was marked as less reliable due to the underlying genomic context (a.k.a. “blacklisted”) [95]. CNVs were assigned a qualitative confidence score (high, medium, or low) based on the above filters (see Additional file 2: Figure S8), and only high-confidence and medium-confidence CNVs were considered for genome-wide analyses. Low-confidence CNVs were recorded and retained for future follow-up studies but were not included in any analyses presented in this manuscript.

#### Resolving cxSV sites

All candidate instances of cxSVs (*i.e*. variants involving two or more different distinct SV signatures or three or more breakpoints) were linked if at least one side of two or more paired-end cluster putative breakpoints were separated by no more than the joint clustering distance used in that batch of libraries and involved a cluster shared by at least one sample, or if the clusters were two opposing unmated breakpoints (*e.g*. a candidate inversion junction with only 5’/5’ oriented read pairs and a second candidate inversion junction with only 3’/3’ oriented read pairs) whose separating distance either overlapped with a cn.MOPS CNV segment in at least one shared sample (via BEDTools intersect, reciprocal overlap 50% required) or was otherwise the only parsimonious resolution for both breakpoints after manual scrutiny of both unmated clusters and all discordant individual read mappings near the unresolved breakpoints. All putative complex SV sites were subsequently categorized by a custom shell script. Complex SV subclasses that could be automatically resolved by this process included all combinations of CNV-flanked inversions (delINV, INVdel, dupINV, INVdup, delINVdel, dupINVdup, delINVdup, dupINVdel), interspersed duplications (iDUP and iDUPdel), and inverted tandem repeats (IR). All computationally predicted complex variants were then manually inspected and revised if necessary. All remaining unresolved putative complex sites were manually investigated where there was evidence of at least six anomalous read-pairs in support per sample, the event appeared in less than 30% of all libraries, or the event featured overlapping paired-end clustering and read-depth CNV segments. All sites unable to be resolved manually or computationally were emitted from the overall SV pipeline as incompletely resolved sites (IRS).

#### SV callset curation

All SV calls output by *Holmes* were subjected to manual inspection to ensure a high-confidence final SV callset. All canonical inversions ≥4 kb, translocational insertions ≥ 4 kb, canonical CNVs ≥ 100 kb, chromosomal translocations, and cxSV were evaluated. Manual inspections consisted of assessing read pair clusters on mapping quality, plotting read-pair mapping coordinates, and—where applicable—visualizing normalized physical sequencing depth with *CNView* at predicted sites of increased or decreased copy number, resulting in visual confirmation of the proposed structure in >95% of manually inspected observations [96]. Second, since all liWGS libraries were prepared from lymphoblastoid cell line (LCL)-derived DNA, we screened our SV callset for large LCL passaging artifacts. We required all unbalanced SVs ≥ 100 kb with less than 30% coverage by size of our CNV blacklisted regions (see above) that appeared in 1/686 participants to have at least one source of orthogonal validation performed on whole blood-derived DNA (most commonly CMA; see section on SV breakpoint validation, below), resulting in an estimated 26 LCL artifacts that were not present in the blood DNA. We also excluded any balanced rearrangements validated in LCL-derived DNA but not in whole blood-derived DNA due to likely being LCL passaging artifacts (n = 2). It is likely that a comparable subset of smaller SVs observed in this study (< 100 kb) may also be LCL artifacts; however, given the high concordance of the callset when compared to two independent sources of validation from whole blood-derived DNA (see “SV breakpoint validation” below), we do not anticipate remaining LCL artifacts to be numerous.

#### Callset merging across sequencing batches

SV callsets from each batch of liWGS libraries (referred to hereafter as “set 1” (n = 278), “set 2” (n = 229), and “set 3” (n = 201), respectively) were merged using an iterative approach as follows. First, a list of non-redundant SV breakpoints was linked between sets. Breakpoints were linked if their mapping spans had at least 20% overlap between sets and their predicted SV type was concordant. Where multiple breakpoint clusters were putatively linked from within the same set, clusters were preferentially selected if they were classified as “Valid” by our heuristic classifier (see above), then ranked by differences in variant allele frequency from the original breakpoint, selecting the top match among this list from each set. Each breakpoint from each set was only allowed to correspond to one non-redundant merged breakpoint, and each non-redundant merged breakpoint could contain at most one breakpoint from each set. The union of samples represented by all linked clusters was taken to create the consolidated list of unique subjects represented in each non-redundant breakpoint cluster. We scrutinized the outcome of this breakpoint linking procedure and identified only 2 total sites (0.01% of all SVs; 1 cxSV and 1 INS) where two similar SVs were not merged into a single consensus variant based on proximal breakpoint coordinates (Collins2017_INS_459 & Collins2017_INS_460; Collins2017_cxSV_213 & Collins2017_cxSV_214; see Additional file 1). Next, any canonical CNV segments not linked based on read-pair clustering as described were further considered for linking between sets based on reciprocal overlap ≥ 50% by size with another canonical CNV segment from a different set. Where multiple canonical CNV segments were eligible for linking from a single set, the CNV with the greatest reciprocal overlap with the original segment was selected. CNV confidence was reassigned to the merged non-redundant CNV segments based on the highest confidence of any contributing CNV. For all analyses, we excluded canonical CNVs designated as low-confidence (n = 6660; not included in any counts reported in “Results,” “Discussion,” figures, tables, or supplement).

### SV validation experiments

We employed five approaches for validation of SVs detected in this cohort, as detailed below.

#### PCR cloning and sanger sequencing

SV validation was performed on 144 SVs with traditional PCR cloning and Sanger sequencing. Primers for breakpoint cloning and Sanger sequencing were designed with Primer3 run at default parameters [97]. Candidate primers were further screened for degenerate hybridization and non-specific product via BLAT and in silico PCR [98]. Primers were synthesized by Integrated DNA Technologies Inc. (Coralville, IA, USA). PCR products were visualized by gel electrophoresis. Sanger sequencing was conducted by GeneWhiz Inc. (South Plainfield, NJ, USA) and the MGH DNA Core (Boston, MA, USA). Sequence alignment was resolved using UCSC BLAT [98]. PCR and Sanger resequencing was performed for a subset of breakpoints from cases TL009, TL010, and UTR22, but these validation experiments were not included for any performances estimates per the genome-wide SV analyses.

#### CMA analysis

CNV detection from SNP CMA was previously performed on 99.0% (679/686) of sequenced subjects used in genome-wide SV analyses, which has been previously described in detail [36, 99]. In brief, genotyping was performed with the Illumina Omni2.5, 1Mv3, or 1Mv1 arrays. CNVs were detected with the CNVision algorithm, which calculates a joint probability for a variant based on three methods (PennCNV, QuantiSNPv2.3, and GNOSIS) [36, 100, 101]. For the purpose of our analysis, we selected unbalanced SVs most likely to be detected at CMA resolution and thus restricted to the 1170 autosomal SVs with at least one segment of predicted dosage imbalance ≥ 40 kb that also did not have ≥ 30% coverage by size with regions of known dosage biases or low-complexity sequences included in our blacklist used during CNV detection, as described earlier. We assessed overlap between CMA-based CNV segments and our predicted intervals of dosage imbalance from liWGS using BEDTools requiring ≥ 50% coverage by size from CMA CNV calls over the predicted liWGS CNV interval [95]. We considered any SVs with at least one segment of dosage imbalance considered in this analysis that validated in at least one expected sample to represent a true positive SV call.

#### Capture sequencing and analysis

Multiplexed high-throughput validation was conducted by simultaneous breakpoint capture sequencing of 427 predicted SV sites across 96 child–parent trios (288 individuals). Breakpoints were selected to represent all possible SV classes; priority was given to rare variants, those predicted to disrupt genes of interest, and those that did not already have orthogonal validation from CMA analysis or PCR and Sanger sequencing at the time of the capture validation experiment. Targeted capture probes were tiled across 2250 bp, flanking both sides of each breakpoint; probe density was progressively concentrated nearest the expected position of the breakpoint to maximize sequencing depth crossing and directly flanking predicted breakpoints. Degenerate probe sequences (*i.e.* probes with multiple possible hybridization sites in the reference genome) were identified by a combination of the *Jellyfish* k-mer counting algorithm and in silico probe sequence alignment with BWA-mem; all degenerate probes were removed from the capture design [102, 103]. Library capture enrichment was performed using the Agilent (Santa Clara, CA, USA) SureSelect XT system and protocols. Ninety-six pools of three samples were prepared, where each pool contained the DNA from one participant, an unrelated mother, and an unrelated father, where all three individuals in the pool were not predicted to share any breakpoints present in the capture design. These 96 pools were barcoded, multiplexed, and sequenced once with a full lane of single-end 101 bp reads and once with a full lane of paired-end 101 bp on an Illumina HiSeq 2500 at the Broad Institute (Cambridge, MA, USA). Two sets of 12 pools received additional sequencing at single-end 150 bp and single-end 300 bp on the Illumina MiSeq platform at MGH to test the effect of longer read lengths in this capture design. Sequencing data were processed as described previously for liWGS libraries. Across all 96 capture libraries, a total of 6.23 billion reads were generated. Sequences crossing putative SV breakpoints (and thus overall SV validity) were obtained by blindly screening all capture data for high-quality individual non-duplicate reads with a primary alignment flanking one side of the predicted breakpoint and a secondary or supplementary alignment flanking the other side of the predicted breakpoint. All candidate split-read sequences were evaluated manually using BLAT to ensure they did not have any equally parsimonious alignments anywhere else in the genome [98]. A subset of breakpoints showed paired-end clustering support without a split read, which we included if they showed a statistically significant enrichment of paired-end reads relative to predicted reference samples.

#### liWGS versus siWGS overlap

We evaluated the overlap between SV calls from the 39 participants for which previously generated siWGS data were available [104]. We considered two approaches for validating liWGS SV calls from siWGS data. For all completely resolved liWGS SV calls (*i.e*. excluding IRS) appearing in at least one of the 39 participants with near-breakpoint precision (i.e. any call with at least one cluster of anomalous liWGS read pairs; n = 2399), we searched that participants’ corresponding siWGS library within a window of ±5 kb from the liWGS-predicted breakpoint coordinates for any anomalous, non-duplicate, primary aligned siWGS pairs mapping to within the 5 kb windows of the predicted breakpoint. Further, we required the aligned orientation of siWGS pairs to match those of the corresponding liWGS pairs. Windows of 5 kb were chosen as the upper bound of conceivable breakpoint imprecision from liWGS alone. Any SV with one breakpoint supported by ≥ 3 unique siWGS read pairs meeting our criteria in at least one expected sample was considered a true positive liWGS call. When comparing siWGS data against our predicted “invalid” clusters of anomalous liWGS read pairs to estimate false negative rates, we conservatively relaxed these thresholds to ±7.5 kb and ≥ 1 unique siWGS read pair. Second, we evaluated evidence from siWGS sequencing depth for all completely resolved (i.e. excluding IRS) autosomal liWGS SV calls appearing in at least one of the 39 participants with at least one interval of dosage imbalance ≥ 10 kb that had < 30% coverage by our blacklisted CNV loci (n = 585; 514 of which also were considered during siWGS read-pair analysis). For this analysis, we first ran cn.MOPS on siWGS libraries for all 39 participants and their families (mothers, fathers, and one sibling each) from available data [104, 105]. Similar to our application of cn.MOPS during liWGS SV discovery (see above), we ran cn.MOPS on this siWGS dataset at bin sizes of 100 bp, 300 bp, 1 kb, and 3 kb, resulting in minimum CNV call sizes of 300 bp, 900 bp, 3 kb, and 9 kb, respectively. We merged the resultant calls per sample across these three bin sizes to obtain an initial set of depth-based CNV calls for comparison versus liWGS. For each interval of dosage imbalance from liWGS that met our criteria for this analysis, we evaluated coverage of that interval against siWGS cn.MOPS calls from that same participant. Any liWGS call with an interval of ≥ 50% coverage by siWGS cn.MOPS calls in at least one expected sample was considered a true positive liWGS SV call. The total number of non-redundant SVs considered by either read-pair or sequencing depth analyses versus siWGS was 2470.

### liWGS sensitivity analysis versus CMA CNVs

We evaluated the sensitivity of liWGS for detection of high-confidence CNVs reported by CMA. As the resolution of CMA is variable across the genome (for example, based on the probe density at a given locus), we applied filters to the raw CNV calls from CMA on the subset of 99.0% of participants in this study for which CMA CNVs had previously been reported [36, 99]. We thus required CMA CNV calls to be ≥ 25 kb, have < 30% coverage by size versus the CNV blacklist applied during liWGS SV discovery, and have a pCNV ≤ 1 × 10^–9^ as required by the published methods for CMA CNV analyses in these same participants by Sanders et al. [36, 99]. For each CMA CNV meeting these criteria, we compared the CNV interval to the predicted intervals of dosage imbalance from fully resolved liWGS SV calls (including canonical CNVs and also unbalanced cxSVs). We considered a CMA CNV to be successfully detected by liWGS if the CMA CNV interval had ≥ 25% coverage by size from regions of dosage imbalance from that participant’s corresponding liWGS SVs. We did not observe major differences in the outcome when requiring different stringencies of reciprocal overlap (up to ~75%).

### liWGS technical replicate analysis

For 22 participants, we sequenced pairs of technical replicate liWGS libraries to assess the consistency of our SV discovery methods, as described above. Given that pairs of technical replicates varied in coverage, and since depth of coverage can bias sensitivity in many variant detection applications [106], we designated the replicate with fewer total fully resolved SV calls in each pair as the truth library and the second replicate as the test library. For each pair, we evaluated concordance of SV calls as the total number of fully resolved SVs from the truth library detected in the test library divided by the total number of fully resolved SVs in the truth library.

### Comparison to other studies and SV reference databases

We downloaded SV callsets as reported in six recent WGS studies of SV outside the SSC [1, 5, 7, 46–48] and two public SV reference databases [49, 50]. We next decomposed each callset into sets of genomic intervals representing deletion, duplication, inversion, and insertion. For studies where cxSVs were reported as multiple intervals (e.g. a delINVdel reported as two deletion intervals and one inversion interval), we separated those intervals into their respective categories prior to comparisons. For studies where cxSVs were reported only as one single interval with no additional information, we treated that interval as a composite complex interval for sake of comparisons. For classes of SV reported that did not fit into any of these previous categories, we added them to a final “other” SV category. From these cleaned callsets, we compared each of the SVs identified in this study to its respective SV category as well as the “other” SV category. For cxSVs, we compared each rearranged interval identified in our study to its respective category and also compared the entire interval spanned by the cxSV to the complex and “other” categories. We determined two intervals to be concordant if they shared 50% reciprocal overlap by size per BEDTools intersect. cxSVs were considered successfully matched in their entirety if all intervals involved in the rearrangement as identified by liWGS in this study had a matching interval in the comparison datasets. If one or more intervals involved in a cxSV were not matched in any of the reference datasets, we considered that cxSV to have been previously discovered but incompletely characterized.

### Evaluating the relationship between inversion breakpoints and long repetitive sequences

We first annotated all inverted loci involved in complex and canonical SVs excluding insertions against annotated repetitive sequences at least 300 bp in length from RepeatMasker and the UCSC segmental duplication track for human assembly GRCh37 [61, 107]. As liWGS does not provide nucleotide-level precision of breakpoints, and instead usually offers a breakpoint resolution of ~1.5 kb, we drew a conservative window of ±500 bp around each predicted inversion breakpoint and intersected against the set of repetitive elements described above using BEDTools intersect while requiring at least one base of overlap [95]. We next shuffled all inversion intervals across the GRCh37 reference genome with BEDTools shuffle, and did not allow breakpoints to be placed in N-masked reference sequences to avoid artificially depleting our simulated inversions from mappable regions of the genome. Importantly, for each simulated set of inversions, we maintained the original size distribution of inversions derived from the experimental liWGS data. We next repeated the repetitive sequence annotation process for each set of simulated inversions, and calculated empirical *p* values by comparing our observed values against all simulated values. We calculated *p* values for all repeat elements in aggregate, but also considered the four most common repeat families independently: SINEs, LINEs, LTRs, and segmental duplications (Seg. Dup.). Finally, we adjusted *p* values for multiple comparisons using a Benjamini–Hochberg correction.

### Genome-wide SV enrichment tests

To assess our callset for the presence of loci enriched in SV beyond random chance, we first segmented the GRCh37 reference genome into 100 kb contiguous bins. We next removed all bins that had at least 10% covered by the CNV mask applied during SV detection to avoid observing artificially depleted bins due to technical limitations. We further restricted this analysis to autosomes. We then overlaid all SVs discovered in this cohort atop the remaining bins (n = 24,742) and counted the number of SVs per bin. We tabulated counts per bin for all fully resolved SVs (i.e. excluding IRS) as well as counts specific to each major SV class except IRS (DEL, DUP, INS, INV, CTX, cxSV). We next made the null assumptions that large SVs are (1) rare events in the genome (as compared to SNPs or InDels) and (2) that they should follow a random distribution across the genome. Given that these assumptions fit the description of a Poisson point process, similar to the observation of sequencing reads by Lander and Waterman [108], we thus evaluated a Poisson test (λ = mean count of SVs per bin) for the count of SVs per bin to evaluate the alternative hypothesis of enrichment of SVs at the tested loci beyond expectation (*e.g*. hypermutable or repeatedly rearranged loci). We subsequently applied the Benjamini–Hochberg procedure to control FDR and assessed genome-wide significance at q ≤ 0.05. Finally, where multiple 100 kb bins each emerged as significantly enriched for SVs beyond expectation and were not separated by more than a single non-significant 100 kb bin, we merged those bins into one larger locus and assigned the maximum *p* value of any one sub-bin to the larger locus.

### Gene annotation

All completely resolved SVs (i.e. excluding IRS) were evaluated for possible genic overlap by breakpoint comparison with all annotated transcripts from the Ensembl gene annotation GTF for hg19/GRCh37 [109]. Intersections were performed with BEDTools intersect for single-breakpoint variants and BEDTools pairtobed for mutli-breakpoint variants [95]. Deletions were classified as LoF if they altered at least one base from any annotated exon. Duplications were classified as LoF if they duplicated one or more bases from any annotated internal exon (i.e. neither the 5’ UTR, 3’ UTR, first exon, or last exon) without spanning beyond the first or last exon of the gene and were classified as whole-gene copy gain (CG) if the duplication encapsulated an entire annotated transcript. Inversions were classified as LoF if one breakpoint localized to an annotated transcript and the other breakpoint localized outside that transcript or if both breakpoints lay within the same transcript and the interval between the two breakpoints spanned at least one annotated exon. Translocations were considered LoF if either breakpoint lay within an annotated transcript. Given that the resolution of liWGS did not permit exact breakpoint base-pair-scale mapping, we did not consider insertions for LoF or CG gene impacts, but did make note if inserted sequence originated from a gene or if sequence was being inserted into a gene. Complex events were annotated by first decomposing the variant into its constituent SV signatures, then interpreting each SV signature simultaneously with the methodology described above to reach a consensus on the overall genic impact of the rearrangement. All interpretation of genic impact was constructed on a transcript-specific basis for each transcript overlapped by each variant. Where relevant, specific gene lists were adopted by those curated by the laboratory of Daniel MacArthur, which are available online (https://github.com/macarthur-lab/gene_lists).

### Non-coding or positional functional effect annotation

All SVs were evaluated for potential non-coding or positional functional effects. Any SV with breakpoints in two different topologically-associated domains (TADs) per annotations by Dixon et al. were recorded as possibly having a disruptive effect on the regulation of any gene encompassed by the disrupted TAD(s) [110]. Further, all SVs were overlaid atop ENCODE promoter and enhancer annotations from all histone marks (H3K27ac, H3K4me1, H3K4me3, HeK9ac) as previously reported by the ENCODE consortium [111, 112]. Per ENCODE recommendations available on the ENCODE website (https://www.encodeproject.org/), promoter regions were derived by merging histone marks H3K4me3 and H3K9ac, while enhancer regions were derived by merging histone marks H3K27ac, H3K4me1, and H3K9ac. Deletions and duplications were annotated for any overlap with a promoter or enhancer, while at least one breakpoint from an insertion, inversion, or translocation had to lie within a promoter or enhancer to be considered as potentially disruptive.

### Scores of intolerance to LoF variation in healthy individuals

Where available, we considered residual variation intolerance scores (RVIS) and LoF constraint scores (pLI) for each gene in the UCSC RefFlat for GRCh37 [66, 67, 107]. As previously described, pLI measures statistical depletion of truncating (LoF) mutations in healthy individuals beyond what is expected by a model that estimates the background mutation rate of every possible trinucleotide combination in the genome, while RVIS calculates the residual depletion of functional mutations (including both LoF and missense) in healthy individuals per gene beyond what is expected by chance [66, 67]. We used the pLI and RVIS scores from the data released circa 2015 summer corresponding to the data published on 60,706 individuals by the Exome Aggregation Consortium [65]. Per specifications of both groups of authors, we considered a gene to be intolerant to/constrained against functional mutation if it had an RVIS score ≤ 10.0 or a pLI ≥ 0.90.

### Real-time quantitative PCR of *MBD5* and *ACVR2A* transcripts

RNA was extracted from 10^6^ LCL cells, obtained through SFARI from the Coriell Cell Repository at Rutgers University (Camden, NJ, USA), from the participant harboring the *de novo* 675 kb inversion at the 2q23.1/*MBD5 *microdeletion locus and two unrelated individuals selected as controls: one affected and an unaffected mother unrelated to either selected participant. Extractions were performed using TRIzol (Invitrogen) followed by RNeasy kit (Qiagen) column purification. First-strand complementary DNA (cDNA) was synthetized using Verso cDNA Synthesis Kit (ThermoFisher Scientific) from 1 ug of total RNA with oligo(dT), random hexamers, and RNase inhibitor. Real-time quantitative PCR (RT-qPCR) was then performed for messenger RNA expression of *MBD5* and *ACVR2A* as well as *ACTB* as an endogenous control with the following primer sequences:

- *ACVR2A* (exons 2-4, forward): 5′ CTG GTG TTG AAC CGT GTT ATG 3′
- *ACVR2A* (exons 2-4, reverse): 5′ GAT TTG AAG TGG GCT GTG TG 3′
- *ACVR2A* (exons 5-6, forward): 5′ GTT ACA CCT AAG CCA CCC TAT TAC 3′
- *ACVR2A* (exons 5-6, reverse): 5′ GCT TTC CAG ACA CAA CCA AAT C 3′
- *MBD5* (exons 3-4, forward): 5′ CAG ATG GCA ACA GAG GATG T 3′
- *MBD5* (exons 3-4, reverse): 5′ GCA GTG TAA TGG AGG CAG TT 3′
- *MBD5* (exons 7-8, forward): 5′ GTG GCT TGG AAT GTC CTC TT 3′
- *MBD5* (exons 7-8, reverse): 5′ TCT GCG GTT CTC TGT TTC AC 3′
- *ACTB* (exons 5-6, forward): 5′ TGA AGT GTG ACG TGG ACA TC 3′
- *ACTB* (exons 5-6, reverse): 5′ GGA GGA GCA ATG ATC TTG AT 3′

Primers and nuclease-free water were added to the LightCycler® 480 SYBR Green I Master Mix (Roche). All samples of cDNA (diluted 1:10) were run in triplicate in final 20 uL reaction volumes. LightCycler® 480 equipment (Roche) was used followed by the manufacturer’s software for Ct calculation. Relative differences in transcript levels were quantified according to the delta Ct method and normalized to *ACTB*. Standard error of the mean (SEM) was calculated for each sample. Results are expressed as fold-change relative to the endogenous control gene normalized to the average of the two control samples.

## Additional files

Additional file 1: Map of 11,735 SV discovered by liWGS among a cohort of 686 ASD participants from the SSC. (XLSX 737 kb)
Additional file 2: Table S1. Comparison of validation methods and false discovery rate estimates. **Table S2** Genomic element enrichments from contrasts of rare vs common SVs. **Table S3** LoF gene set enrichment statistics from contrasts of rare vs common SVs. **Table S4** Twenty loci with genome-wide significant SV enrichments. **Table S5** Ten loci with genome-wide significant rare SV enrichments. **Table S6** Literature review of previous reports of germline chromoanagenesis. **Table S6** Comparison of three cases of extreme chromoanagenesis. **Figure S1** Example of a cxSV with unknown impact on gene function. **Figure S2** Trends among cxSV subclasses. **Figure S3** Characteristics of canonical and complex inversion variation. **Figure S4** lrWGS resolved and phased an 83.7 kb delINVdel in a participant with ASD. **Figure S5** lrWGS resolved and phased a 97.6 kb dupTRIPdup-INV in a participant with ASD. **Figure S6** Genome-wide significant accumulation of rare SV at 10 loci. **Figure S7** Proposed mutational timeline of chromoanasynthesis in TL009. **Figure S8** Computational framework for consensus classification of canonical CNV segments. **Figure S9** A de novo 14 kb deletion of VRK1 in a participant with ASD. **Figure S10** A de novo inversion of the 2q23.1 syndrome locus in a participant with ASD. **Supplemental Results 1:** Evaluation of SV discovery methods from six independent validation methods. **Supplemental Results 2:** Coding and noncoding functional annotations for all fully resolved SVs. **Supplemental Results 3:** Genome-wide SV aggregation analyses. **Supplemental Results 4:** Cryptic de novo SV that may contribute to ASD risk. **Supplemental Note 1.** Definition and validation of cxSVs. (PDF 3.57 mb)
Additional file 3: Rearrangement details for chromoanagenesis in participants TL009, TL010, and UTR22. (XLSX 59 kb)
Additional file 4: List of IDs and accession codes for the 686 participants selected from the SSC. (TXT 15 kb)

## Abbreviations

ASD: Autism spectrum disorder
CMA: Chromosomal microarray
CNV: Copy-number variation
cxSV: Complex structural variation
liWGS: Long-insert whole-genome sequencing
LoF: Loss-of-function
lrWGS: Linked-read whole-genome sequencing (10X Genomics)
NDD: Neurodevelopmental disorder
siWGS: Short-insert whole-genome sequencing
SV: Structural variation
VF: Variant frequency
WES: Whole-exome sequencing
WGS: Whole-genome sequencing
