# Supplementary material for: Defining the diverse spectrum of inversions, complex structural variation, and chromothripsis in the morbid human genome
Source: Genome Biol. 2017 Mar 6;18:36. doi: 10.1186/s13059-017-1158-6 (PMC5338099; doi:10.1186/s13059-017-1158-6)
Supplement: Additional file 2: Table S1. — Comparison of validation methods and false discovery rate estimates. Table S2 Genomic element enrichments from contrasts of rare vs common SVs. Table S3 LoF gene set enrichment statistics from contrasts of rare vs common SVs. Table S4 Twenty loci with genome-wide significant SV enrichments. Table S5 Ten loci with genome-wide significant rare SV enrichments. Table S6 Literature review of previous reports of germline chromoanagenesis. Table S6 Comparison of three cases of extreme chromoanagenesis. Figure S1 Example of a cxSV with unknown impact on gene function. Figure S2 Trends among cxSV subclasses. Figure S3 Characteristics of canonical and complex inversion variation. Figure S4 lrWGS resolved and phased an 83.7 kb delINVdel in a participant with ASD. Figure S5 lrWGS resolved and phased a 97.6 kb dupTRIPdup-INV in a participant with ASD. Figure S6 Genome-wide significant accumulation of rare SV at 10 loci. Figure S7 Proposed mutational timeline of chromoanasynthesis in TL009. Figure S8 Computational framework for consensus classification of canonical CNV segments. Figure S9 A de novo 14 kb deletion of VRK1 in a participant with ASD. Figure S10 A de novo inversion of the 2q23.1 syndrome locus in a participant with ASD. Supplemental Results 1: Evaluation of SV discovery methods from six independent validation methods. Supplemental Results 2: Coding and noncoding functional annotations for all fully resolved SVs. Supplemental Results 3: Genome-wide SV aggregation analyses. Supplemental Results 4: Cryptic de novo SV that may contribute to ASD risk. Supplemental Note 1. Definition and validation of cxSVs. (PDF 3.57 mb) [file 13059_2017_1158_MOESM2_ESM.pdf]

Additional File 2: Supplemental Tables & Figures

Defining the Diverse Spectrum of Inversions, Complex Structural Variation, and Chromothripsis in the Morbid Human Genome

Ryan L. Collins, Harrison Brand, Claire E. Redin, Carrie Hanscom, Caroline Antolik, Matthew R. Stone, Joseph T. Glessner, Tamara Mason, Giulia Pregno, Naghmeh Dorrani, Giorgia Mandrile, Daniela Giachino, Danielle Perrin, Cole Walsh, Michelle Cipicchio, Maura Costello, Alexei Stortchevoi, Joon-Yong An, Benjamin B. Currall, Catarina M. Seabra, Ashok Ragavendran, Lauren Margolin, Julian A. Martinez-Agosto, Diane Lucente, Brynn Levy, Stephan J. Sanders, Ronald J. Wapner, Fabiola Quintero-Rivera, Wigard Kloosterman, Michael E. Talkowski

Table of Contents

Item *Page*

Supplemental Table 1 .... S-2
Supplemental Table 2 .... S-3
Supplemental Table 3 .... S-4
Supplemental Table 4 .... S-5
Supplemental Table 5 .... S-6
Supplemental Table 6 .... S-7
Supplemental Table 7 .... S-8

Supplemental Figure 1 .... S-9
Supplemental Figure 2 .... S-10
Supplemental Figure 3 .... S-11
Supplemental Figure 4 .... S-12
Supplemental Figure 5 .... S-13
Supplemental Figure 6 .... S-14
Supplemental Figure 7 .... S-15
Supplemental Figure 8 .... S-16
Supplemental Figure 9 .... S-17
Supplemental Figure 10 .... S-18

Supplemental Results 1 .... S-19
Supplemental Results 2 .... S-20
Supplemental Results 3 .... S-21
Supplemental Results 4 .... S-22

Supplemental Note 1 .... S-23

Supplemental References S-24

Supplemental Table 1. Comparison of validation methods and false discovery rate estimates.

All Validation Sources (as reported in study)

Variant Class	Attempted	Validated	Failed	PPV	FDR
DEL	2,125	2,013	112	94.7%	5.3%
DUP	822	657	165	79.9%	20.1%
INV	244	219	25	89.8%	10.2%
INS	407	314	93	77.1%	22.9%
CTX	4	3	1	75.0%	25.0%
CPX	154	150	4	97.4%	2.6%
ALL	3,756	3,356	400	89.4%	10.6%

PCR/Sanger, Targeted Capture, and CMA (no siWGS)

Variant Class	Attempted	Validated	Failed	PPV	FDR
DEL	681	618	63	90.7%	9.3%
DUP	591	461	130	78.0%	22.0%
INV	42	38	4	90.5%	9.5%
INS	41	31	10	75.6%	24.4%
CTX	4	3	1	75.0%	25.0%
CPX	95	91	4	95.8%	4.2%
ALL	1,454	1,242	212	85.4%	14.6%

siWGS Alone (no PCR/Sanger, Targeted Capture, or CMA)

Variant Class	Attempted	Validated	Failed	PPV	FDR
DEL	1,522	1,461	61	96.0%	4.0%
DUP	296	235	61	79.4%	20.6%
INV	209	187	22	89.5%	10.5%
INS	372	287	85	77.2%	22.8%
CTX	0	0	0	NA	NA
CPX	71	67	4	94.4%	5.6%
ALL	2,470	2,237	233	90.6%	9.4%

PPV: positive predictive value; FDR: false discovery rate

Supplemental Table 2. Genomic element enrichments from contrasts of rare vs common SV.

		Fraction of SV Disrupting Element						
		Rare SV		Common SV			Fisher's Exact Test	
**Element**	**Citation(s)**	Fraction of Rare SV (n=8,323)	95% CI	Fraction of Common SV (n=2,785)	95% CI	**Rare:Common N-Fold Enrichment**	p	OR
Genes	Rosenbloom (2015)	0.281	± 0.010	0.236	± 0.016	1.193	1.30E-06	1.269
Promoters	Thurman (2012) ENCODE (2012)	0.345	± 0.010	0.200	± 0.015	1.720	6.12E-49	2.099
Enhancers	Thurman (2012) ENCODE (2012)	0.346	± 0.010	0.210	± 0.015	1.645	6.04E-43	1.985
TADs	Dixon (2012)	0.023	± 0.004	0.012	± 0.004	1.889	3.98E-04	1.909

CI: confidence interval; OR: odds ratio

Supplemental Table 3. LoF gene set enrichment statistics from contrasts of rare vs common SV.

		Fraction of Genes Disrupted by ≥ 1 LoF SV						
		Rare SV		Common SV			Fisher's Exact Test	
**Gene Set**	**Citation(s)**	Fraction of Genes w/LoF from Rare SV (n=1,334)	95% CI	Fraction of Genes w/LoF from Common SV (n=318)	95% CI	**Rare:Common N-Fold Enrichment**	p	OR
Intolerant to Functional Mutation	Petrovski (2013)	0.110	± 0.018	0.065	± 0.030	1.695	1.34E-02	1.780
Intolerant to LoF	Samocha (2014) Lek (2016)	0.138	± 0.019	0.088	± 0.032	1.567	8.91E-03	1.657
Exonic Deletion Burden in Clinical NDD Cases	Talkowski (2012)	0.157	± 0.019	0.076	± 0.028	2.059	2.48E-05	2.256
≥1 Reported Pathogenic Mutation in ClinVar	Landrum (2014)	0.138	± 0.016	0.077	± 0.025	1.801	1.14E-04	1.928
Autosomal Dominant Disease Locus	Blekhman (2008) Berg (2013)	0.031	± 0.008	0.015	± 0.011	2.110	3.05E-02	2.146

CI: confidence interval; OR: odds ratio

Supplemental Table 4. 20 loci with genome-wide significant SV enrichments.

Chr°	Start (Mb)	End (Mb)	Size (kb)	Cytoband	SVs	Unadjusted p†	B-H Adjusted q*	Genes
3	1.9	2.0	100	3p26.3	6	1.41E-06	3.48E-02	-
3	4.0	4.3	300	3p26.1	7	9.20E-08	2.28E-03	-
4	0.1	0.2	100	4p16.3	9	2.80E-10	6.94E-06	*ZNF718*
4	80.8	80.9	100	4q21.21	6	1.41E-06	3.48E-02	*ANTXR2*
5	39.7	39.8	100	5p13.1	6	1.41E-06	3.48E-02	-
6	13.1	13.2	100	6p24.1	6	1.41E-06	3.48E-02	*PHACTR1*
6	95.5	95.6	100	6q16.1	7	9.20E-08	2.28E-03	-
6	162.7	162.8	100	6q26	6	1.41E-06	3.48E-02	*PARK2*
7	111.0	111.3	300	7q31.1	8	5.35E-09	1.32E-04	*IMMP2L*
8	47.0	47.4	400	8q11.1	8	5.35E-09	1.32E-04	-
9	11.7	11.8	100	9p23	6	1.41E-06	3.48E-02	-
10	68.2	68.5	300	10q21.3	9	2.80E-10	6.94E-06	*CTNNA3*
10	124.3	124.4	100	10q26.13	7	9.20E-08	2.28E-03	*DMBT1*
11	1.9	2.0	100	11p15.5	6	1.41E-06	3.48E-02	*LSP1, TNNT3, MRPL23*
12	38.3	38.4	100	12q12	6	1.41E-06	3.48E-02	-
13	82.3	82.4	100	3q31.1	6	1.41E-06	3.48E-02	-
14	23.1	23.2	100	14q11.2	8	5.35E-09	1.32E-04	-
15	22.9	23.1	200	15q11.2	9	2.80E-10	6.94E-06	*CYFIP1, NIPA1, NIPA2*
20	14.7	14.9	200	20p12.1	7	9.20E-08	2.28E-03	*MACROD2*
20	41.2	41.3	100	20q12	6	1.41E-06	3.48E-02	*PTPRT*

°All coordinates reported from GRCh37

†p-values calculated from the Poisson distribution; see Methods

*Benjamini-Hochberg correction procedure

Supplemental Table 5. 10 loci with genome-wide significant rare SV enrichments.

Chr°	Start (Mb)	End (Mb)	Size (kb)	Cytoband	Rare SVs	Unadjusted p†	B-H Adjusted q*	Genes
3	1.9	2.0	100	3p26.3	6	3.35E-07	8.28E-03	-
3	4.1	4.3	200	3p26.1	7	1.76E-08	4.36E-04	-
6	95.5	95.6	100	6q16.1	7	1.76E-08	4.36E-04	
6	162.7	162.8	100	6q26	6	3.35E-07	8.28E-03	*PARK2*
7	111.0	111.3	300	7q31.1	7	1.76E-08	4.36E-04	*IMMP2L*
8	47.0	47.4	400	8q11.1	7	1.76E-08	4.36E-04	-
9	11.7	11.8	100	9p23	6	3.35E-07	8.28E-03	-
10	68.2	68.5	300	10q21.3	7	1.76E-08	4.36E-04	*CTNNA3*
15	22.9	23.1	200	15q11.2	9	3.48E-11	8.61E-07	*CYFIP1, NIPA1, NIPA2*
20	41.2	41.3	100	20q12	6	3.35E-07	8.28E-03	*PTPRT*

°All coordinates reported from GRCh37
†p-values calculated from the Poisson distribution; see Methods
*Benjamini-Hochberg correction procedure

Supplemental Table 6. Literature review of previous reports of germline chromoanagenesis.

First Author	Year	Journal	Total Chromoanagenesis Rearrangements: Affected	Total Chromoanagenesis Rearrangements: Healthy	Balanced[1] Chromothripsis: Affected	Balanced[1] Chromothripsis: Healthy	Unbalanced[1] Chromothripsis: Affected	Unbalanced[1] Chromothripsis: Healthy	Chromoanasynthesis: Affected	Chromoanasynthesis: Healthy	Chromoplexy: Affected	Chromoplexy: Healthy	Notes
Kloosterman	2011	*Hum Mol Genet*	1	0	1	0	0	0	0	0	0	0	
Liu[2]	2011	*Cell*	4	0	0	0	0	0	4	0	0	0	Report 17 cases of suspected cxSVs, 4 of which meet proposed criteria for chromoanagenesis
Chiang[2]	2012	*Nat Genet*	4	0	4	0	0	0	0	0	0	0	Report 10 cases of cxSVs, 4 of which meet proposed criteria for chromoanagenesis
Kloosterman[2]	2012	*Cell Rep*	7	0	3	0	4	0	0	0	0	0	Report 10 cases of cxSVs, 7 of which meet proposed criteria for chromoanagenesis
Nazaryan	2013	*Eur J Hum Genet*	1	0	1	0	0	0	0	0	0	0	
Plaisancie	2014	*Eur J Med Genet*	1	0	0	0	0	0	1	0	0	0	
Zanardo	2014	*Mol Genet Genom*	1	0	0	0	0	0	1	0	0	0	
de Pagter	2015	*Am J Hum Genet*	0	3	0	3	0	0	0	0	0	0	Children of all three healthy controls manifested severe phenotypes due to *de novo* rearrangements (CNVs) arising at chromothripsis breakpoints from parents
Macera	2015	*Prenat Diagn*	1	0	0	0	0	0	0	0	1	0	
Weckselblatt[2]	2015	*Genome Res*	3	0	1	0	1	0	0	0	1	0	
Bertelson	2016	*Genet in Med*	0	1	0	1	0	0	0	0	0	0	
Masset°	2016	*Hum Mutat*	3	0	0	0	0	0	3	0	0	0	
Redin[2]	2016	*Nat Genet*	27	0	13	0	10	0	0	0	4	0	Report 38 rearrangements that meet proposed criteria for chromoanagenesis, 27/38 of which were not reported by other studies in this table. Previously reported cases were not included in counts here.
Present Study			*2*	*0*	*1*	*0*	*0*	*0*	*1*	*0*	*0*	*0*	*Case UTR22 also included in Redin, 2016*
**TOTAL HUMAN GERMLINE CASES**			**55**	**4**	**24**	**4**	**15**	**0**	**10**	**0**	**6**	**0**	

1: Rearrangement balance defined as net copy loss and copy gain totalling to < 5% of all aggregate rearranged bases

2: When the authors' original classification conflicted with our proposed criteria, we considered the events as they would be classified under our definitions. Counts may therefore not match those reported in the original publication.

Supplemental Table 7. Comparison of three cases of extreme chromoanagenesis.

Case ID	TL010	UTR22[1]	TL009
**Class**	Chromothripsis	Chromothripsis	Chromoanasynthesis
**Chromosomes**	4	4	1
**Breakpoints**	65	40	9
**Fragments**	61	36	8
**CNV Segments**	5	1	9
**Bases Rearranged (Total)**	60.6 Mb	41.5 Mb	53.7 Mb
**Bases Rearranged (Unbalanced)**	840.7 kb	361.2 kb	19.5 Mb
**% Unbalanced**	1.4%	0.9%	36.3%
**Genes Truncated (LoF)**	29	13	12
**Genes Duplicated (CG)**	0	0	567
**Origin**	*De novo* in germline	*De novo* in germline	Likely mosaic[2]

1: Recently reported in Redin *et al.*, *Nat. Genet.*, 2016

2: See **Additional File 2: Supplemental Figure 7**

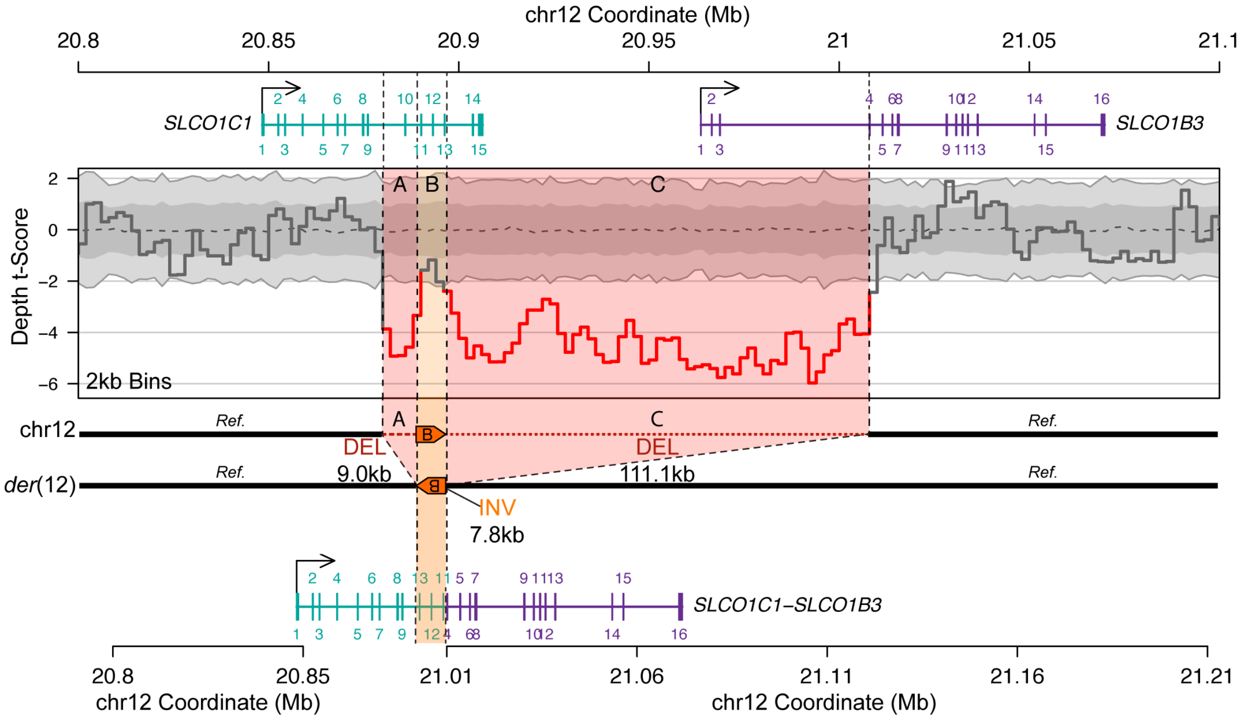

Supplemental Figure 1. Example of a cxSV with unknown impact on gene function. A 127.9 kb complex paired-deletion inversion ("delINVdel") alters multiple loci and may lead to a complex fusion product, *SLCO1C1-SLCO1B3,* including the net loss of five exons and inversion of three exons. *SLCO1C1* has been previously described in ASD due to a *de novo* truncating coding mutation in an ASD-affected subject [1], and one component of this cxSV, a flanking deletion, was previously identified by WES [2]. liWGS sequencing depth visualization was generated with CNView [3].

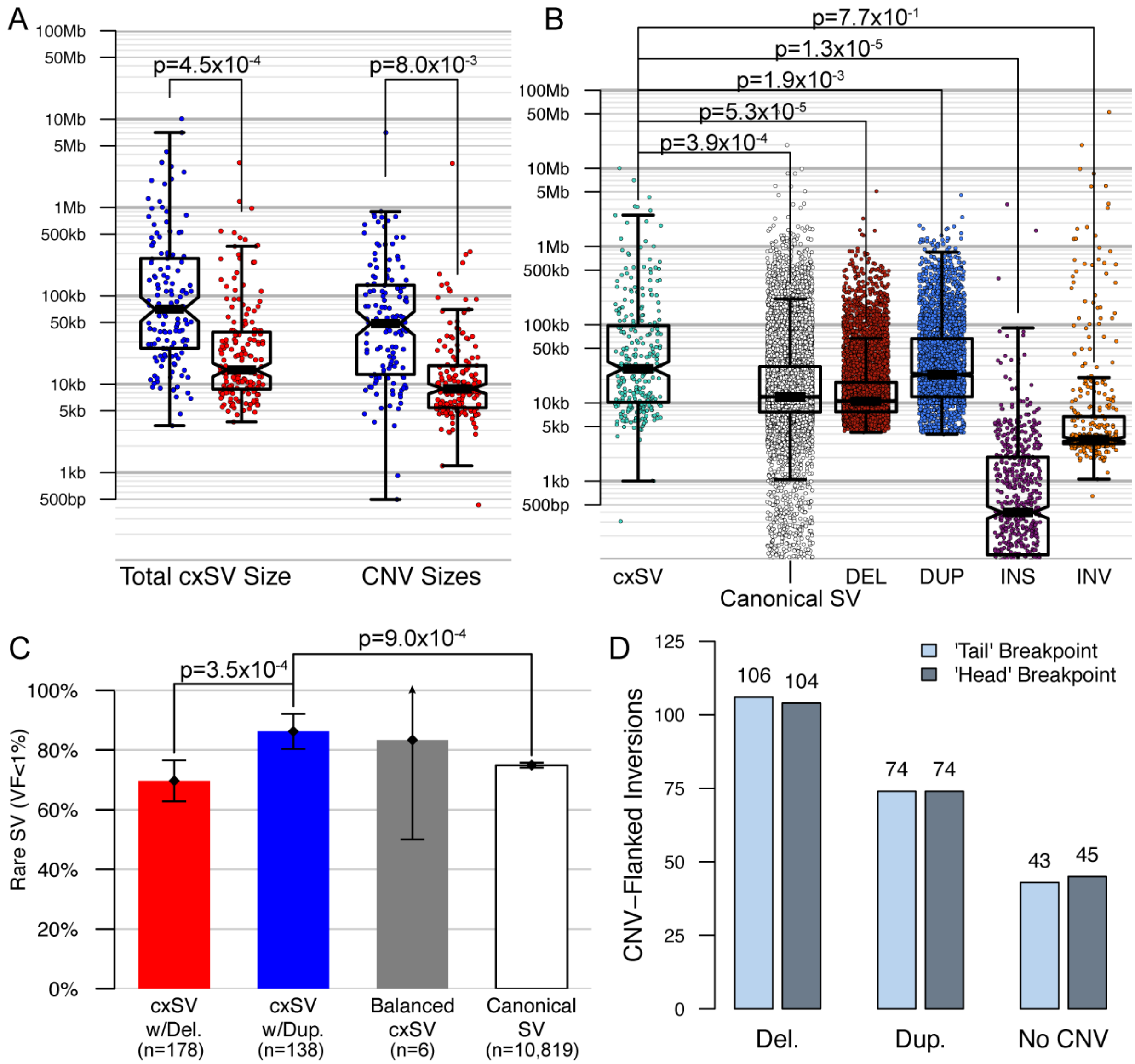

Supplemental Figure 2. Trends among cxSV subclasses. (**A**) Left: cxSV involving duplication (blue) were significantly larger than cxSV involving deletion (red) ($p=4.54x10^{-4}$; one-tailed Welch's t-test (WTT)). Right: duplicated segments involved in all cxSV (blue) were significantly larger than deleted segments involved in all cxSV (red) ($p=7.99x10^{-3}$; one-tailed WTT). (**B**) cxSV were larger on average than all canonical SV collectively as well as each individual canonical SV subclass except for inversions. All comparisons were performed on untransformed SV sizes (note: log_{10} sizes plotted here) and were evaluated with one-tailed WTTs. (**C**) cxSV involving duplication (blue) were significantly rarer in this cohort than either cxSV involving deletion (red; $p=3.49x10^{-4}$) or all canonical SV (white; $p=8.96x10^{-4}$). (**D**) Among complex CNV-flanked inversions, which comprised the majority of all cxSV, there was no significant bias for CNVs to arise at either the 'head' (*i.e.* 5':5' junction) or 'tail' (*i.e.* 3':3' junction) inversion breakpoints. This observation was consistent when considering flanking deletions alone, flanking duplications alone, or all flanking CNVs collectively.

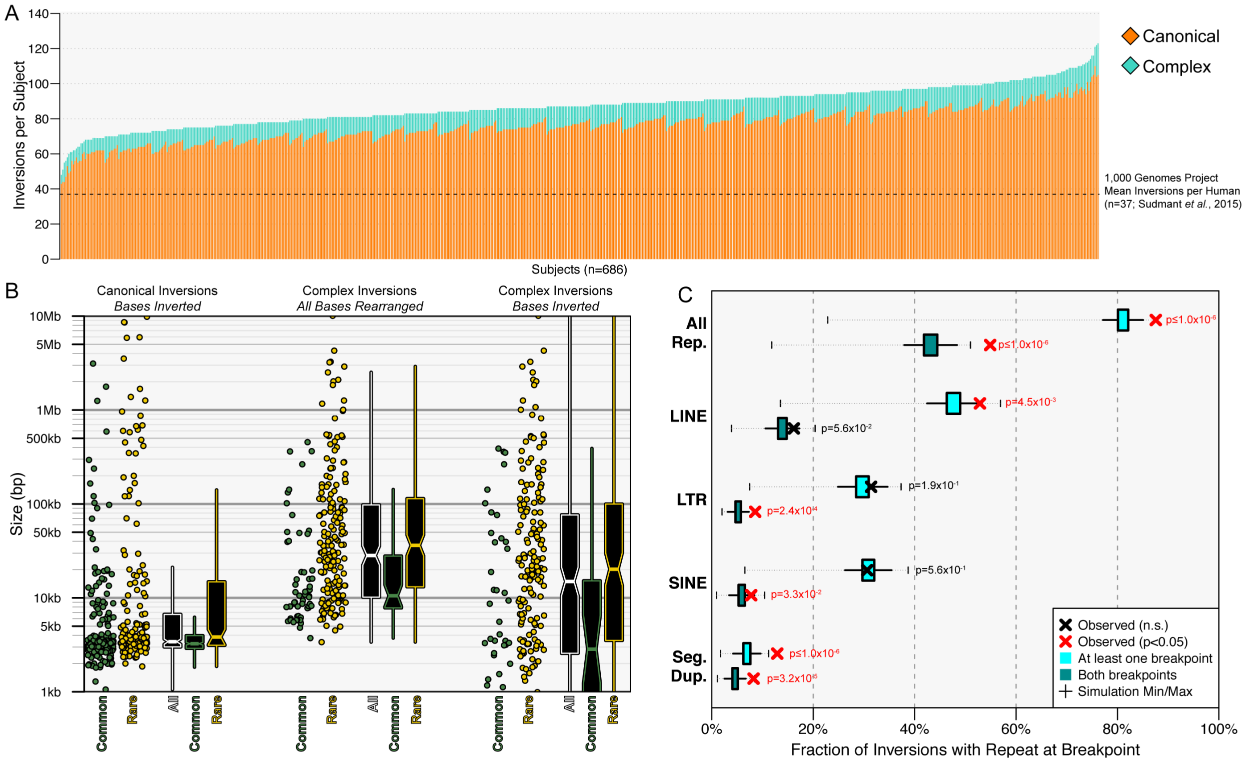

Supplemental Figure 3. Characteristics of canonical and complex inversion variation. (**A**) Totals of canonical (orange) and complex (teal) inversion variants observed per subject and comparison to results from the 1,000 Genomes Project, which reported 37 inversion alleles per genome across 2,504 individuals [4]. liWGS analyses in these ASD probands identified a median of 76 canonical & 11 complex inversions per genome with validation rates of 89.8% (canonical inversions) and 96.9% (complex inversions). (**B**) Complex inversions (center & right) were significantly larger than canonical inversions (left), both in bases rearranged (center) and bases inverted (right). Rare variants (yellow) were larger than common variants (green) for both canonical and complex inversions. (**C**) Inversion breakpoints were enriched in long (≥300bp) repetitive sequences per RepeatMasker for GRCh37, and especially segmental duplications (Seg. Dup.), as compared to the null distribution of one million matched simulations (boxplots).

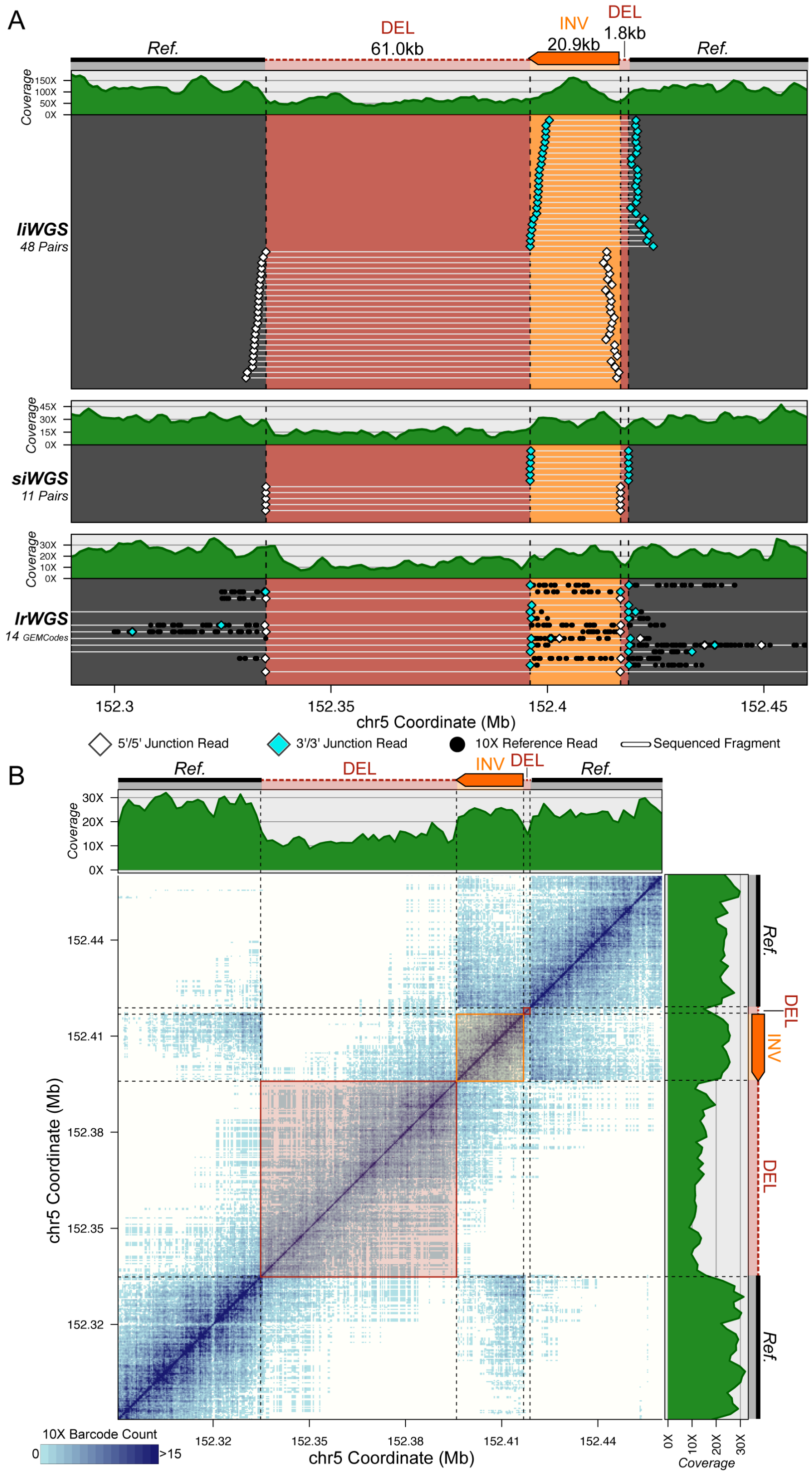

Supplemental Figure 4. lrWGS resolved and phased an 83.7kb delINVdel in a subject with ASD. (**A**) Coverage tracks (green) and sequenced fragments (white) for liWGS (top), siWGS (middle), and lrWGS (bottom) all provided evidence for an inversion flanked by two deletions. All three WGS approaches also had anomalous read-pair support for 5'/5' (white) and 3'/3' (teal) inversion junctions. (**B**) A lrWGS "read-cloud" barcode heatmap, which illustrates the count of shared 10X barcodes between pairs of coordinates on the X and Y axes, also clearly showed evidence for linked molecules passing over both deletions (red boxes) and into the inversion (orange box). Breakpoint coordinates are delineated with dashed black lines; coverage is again provided in green as per panel (**A**). This heatmap was generated with the 10X Loupe software (https://www.10xgenomics.com/software/) [5].

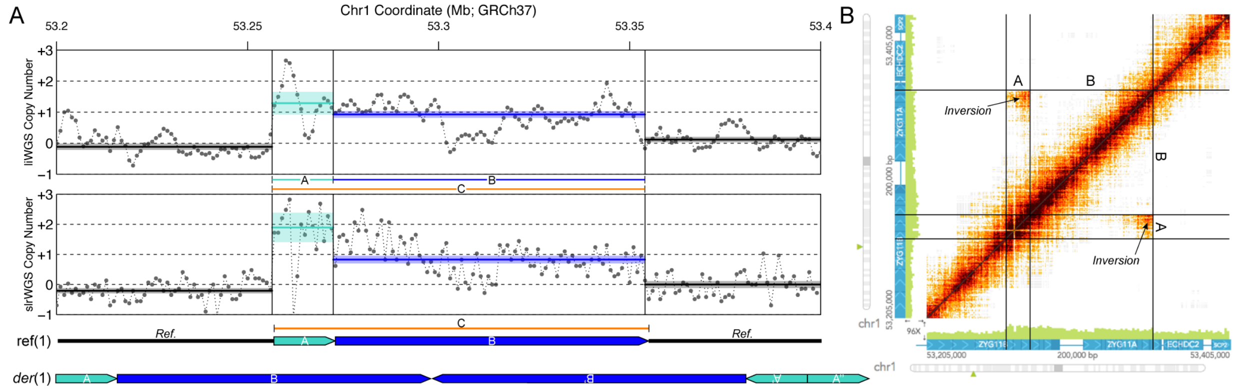

Supplemental Figure 5. lrWGS resolved and phased a 97.6kb dupTRIPdup-INV in a subject with ASD. (**A**) Changes in copy state estimated by liWGS (top) and lrWGS (bottom) depth of coverage confirmed the predicted segment of triplication, labeled "A" (teal), and segment of duplication, labeled "B" (blue), involved in this complex unbalanced inversion, labeled "C" (orange). Solid horizontal lines represent the mean copy state across the marked interval and shaded backgrounds correspond to 95% confidence intervals. (**B**) A lrWGS "read-cloud" barcode heatmap showed evidence for both the duplicated and triplicated segments (coverage tracks, green) and inversion breakpoints (marked with arrows). This heatmap was generated with the 10X Loupe software (https://www.10xgenomics.com/software/) [5].

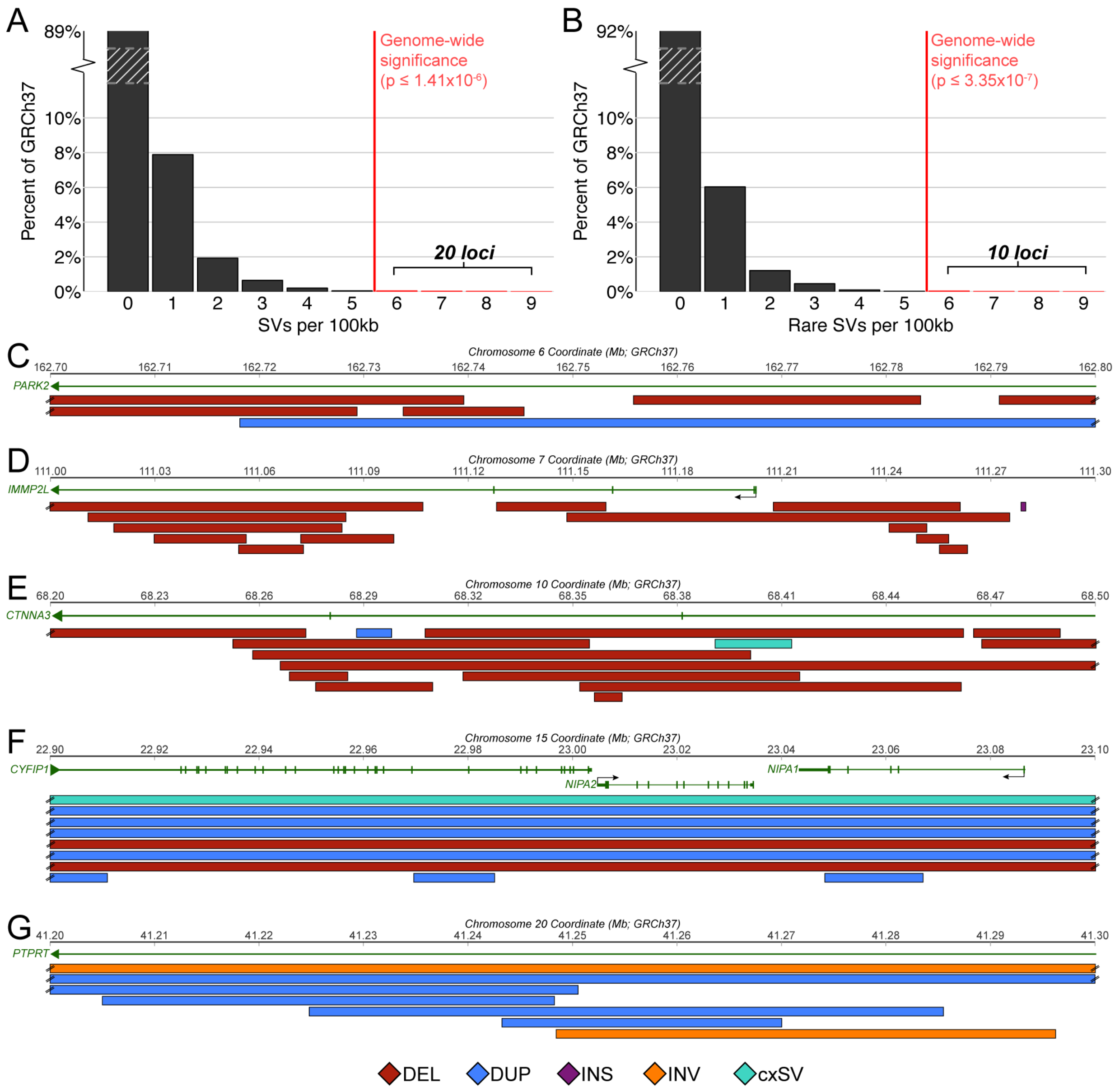

Supplemental Figure 6. Genome-wide significant accumulation of rare SV at 10 loci. We assessed the distribution of all SVs across the genome to screen for any loci enriched in SV beyond expectation (**Additional File 2: Supplemental Results 3**). (**A**) Genome-wide distribution of all SVs per 100 kb bin. Significance was assessed from a Poisson distribution after Benjamini-Hochberg correction ($\alpha \leq 0.05$). Twenty loci were significant by this approach. (**B**) Genome-wide distribution of rare (VF<1%) SV per 100 kb bin. Significance was assessed with the same method as for all SV when unrestricted on VF. Ten loci were significant by this approach, five of which overlapped genes and are highlighted in this figure. (**C**) Rare SV hotspot at 6q26 (*PARK2*; p=3.35x10^{-7}; q=8.28x10^{-3}). (**D**) Rare SV hotspot at 7q31.1 (*IMMP2L*; p=1.76x10^{-8}; q=4.36x10^{-4}). (**E**) Rare SV hotspot at 10q21.3 (*CTNNA3*; p=1.76x10^{-8}; q=8.61x10^{-7}). (**F**) Rare SV hotspot at 15q11.2 (*CYFIP1*, *NIPA1*, *NIPA2*; p=3.48x10^{-11}; q=4.36x10^{-4}). (**G**) Rare SV hotspot at 20q12 (*PTPRT*; p=3.35x10^{-7}; q=8.28x10^{-3}).

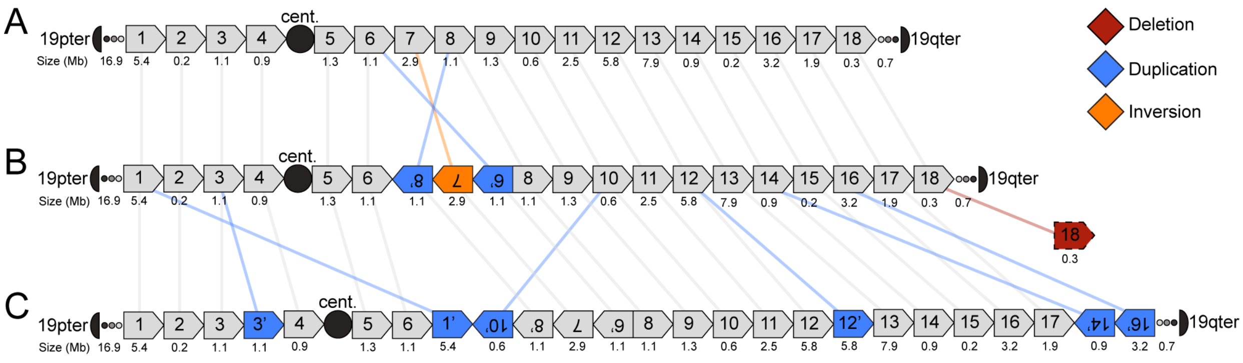

Supplemental Figure 7. Proposed mutational timeline of chromoanasynthesis in TL009. liWGS analysis suggested that the complex rearrangement observed on chromosome 19 in subject TL009 may not have arisen in a single mutational event. A potential model by which the rearrangement could have occurred is provided here. Colored segments demark rearrangements newly arising in each stage. (**A**) Normal chromosome 19 in maternal germline. (**B**) An initial 5.1 Mb dupINVdup (segments 6-8) arose either *de novo* in the maternal germline or early in embryonic development, resulting in observed copy states of nearly three copies from liWGS (2.93 ± 0.08 and 2.83 ± 0.09 copies for segments 6 and 8, respectively; 95% confidence intervals). (**C**) A subsequent mutational event later in development may have then occurred involving widespread reorganization of the same maternal homologue. Copy states for segments 1, 3, 10, 12, 14, and 16 are all significantly lower than both segments 6 and 8, and were estimated at approximately 2.57 ± 0.02 copies from liWGS (95% confidence interval). These data support the possibility of this second mutational event involving mitotic missegregation into a micronucleus and subsequent chromoanasynthesis, as has been previously proposed as a primary mechanism for chromoanagenesis [6-9], possibly attributable to instability in the presence of the large *de novo* dupINVdup arising within 2.4 Mb of the centromere.

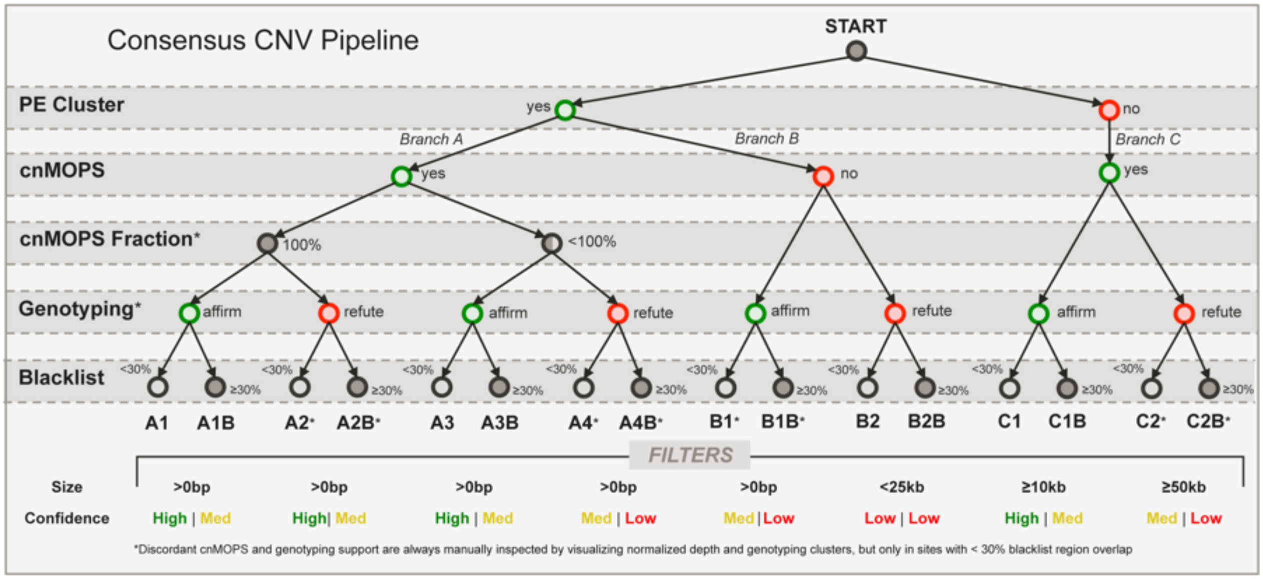

Supplemental Figure 8. Computational framework for consensus classification of canonical CNV segments. We used a tiered pipeline to integrate physical depth and paired-end (PE) clustering SV signals from liWGS data, and to score groups of similar putative CNV calls for qualitative confidence in each call. In brief: we considered PE clustering and physical depth calls both jointly where they overlapped as well as independently where they did not. After refining a set of all candidate CNV intervals, we applied a k-means copy-state genotyping algorithm to affirm or refute each interval. Finally, we screened CNVs on overlap with a set of blacklist regions including poorly mapping sequences, N-masked reference bases, and known multicopy loci (e.g. segmental duplications/low-copy repeats). See **Methods** for a full description of this tiered integration process. Only CNVs scored as "high" or "medium" confidence were included for genome-wide analyses, while "low" confidence CNV calls were excluded from all analyses, counts, and statistics.

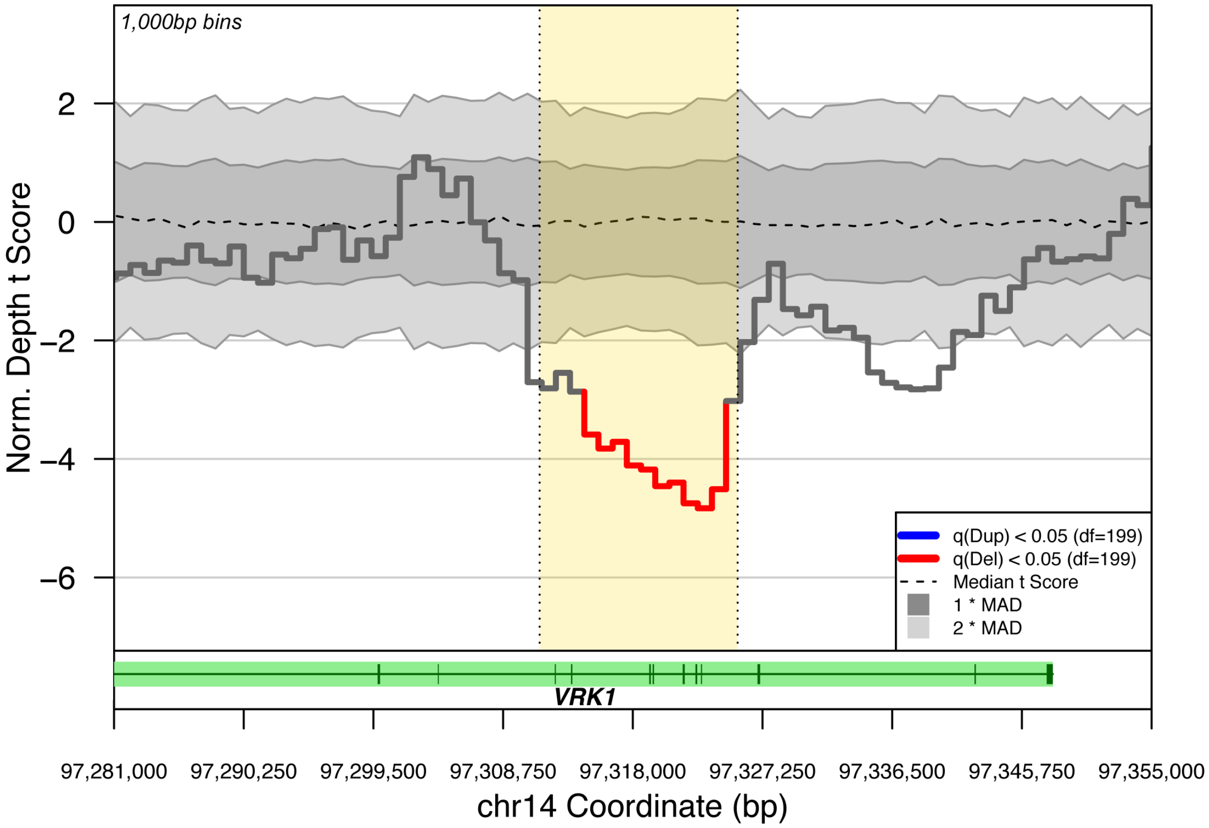

Supplemental Figure 9. A *de novo* 14kb deletion of *VRK1* in a subject with ASD. liWGS analysis in a subject with ASD discovered a *de novo* 14kb deletion (yellow) of seven exons (green) at the vaccinia-related kinase 1 (*VRK1*) locus. *VRK1* had not been directly implicated in ASD by genetic evidence previously, but is coexpressed with known ASD risk factors [10], is involved in neuronal migration and axon maintenance [11, 12], is a chromatin remodeling factor [13], and was predicted to be the most interconnected gene in the genome with other genes recurrently mutated in ASD [14]. The liWGS sequencing depth visualization shown here was generated with CNView [3]. This *de novo* deletion was not previously ascertained by CMA or WES analyses [15, 16], illustrating the value of WGS for SV in ASD and other human disease phenotypes with partial genetic etiologies.

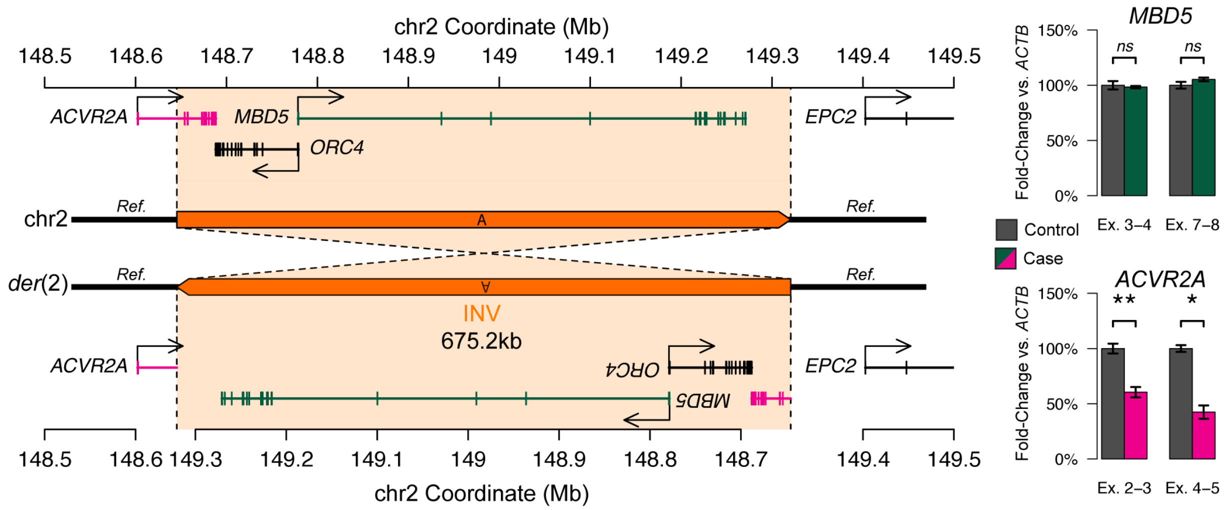

Supplemental Figure 10. A *de novo* inversion of the 2q23.1 syndrome locus in a subject with ASD. liWGS analysis in a subject with ASD discovered a *de novo* 675.2kb inversion of the 2q23.1/*MBD5* microdeletion syndrome locus, which completely inverted the known driver gene of 2q23.1 syndrome, *MBD5*, yet had no observable functional effect on *MBD5* expression in proband lymphoblasts (green barplots; exons 3-4 and 7-8 tested by RT-qPCR). Direct disruption of *ACVR2A* resulted in reduced expression (pink barplots; exons 2-3 and 4-5 tested by RT-qPCR). Both *MBD5* and *ACVR2A* are constrained against truncating coding variation [17, 18], and both genes also lie within the 2q23.1 microdeletion syndrome critical region [19-21]. This inversion provides evidence for a possible secondary contributing effect from *ACVR2A* in the NDD phenotypes associated with 2q23.1 syndrome, and further highlights the value of WGS for SV in studies of disease genomics.

Supplemental Results 1: Evaluation of SV discovery methods from six independent validation methods. We attempted validation of 33.8% (3,756/11,108) of all fully resolved SVs using five orthogonal approaches, including: (1) PCR and Sanger sequencing of breakpoints (n=114 SV tested), (2) comparison to chromosomal microarray (CMA) data available for 99.0% (679/686) of subjects (n=1,170 SV tested) [15, 22], (3) multiplexed targeted breakpoint capture and deep resequencing in 96 subjects and their parents (n=230 SV tested), (4) linked-read WGS (n=3 SV tested), and (5) comparison to standard short-insert whole-genome sequencing (siWGS) data available on a subset of subjects (n=39 subjects; n=2,470 SV tested) [23]. From these experiments, 89.4% (3,356/3,756) of all assessed SVs were validated by at least one method (**Figure 1C** and **Additional File 1**). We also sequenced pairs of technical replicate liWGS libraries in 22 subjects to further appraise our methods. Based on these replicate library pairs, we found a median of 87.5% concordance between replicate libraries. We attempted to estimate a false negative rate by evaluating 32,472 low-quality candidate SV read clusters that were filtered as 'invalid' by our computational SV pipeline, finding that 5.9% (1,906/32,472) of computationally predicted 'invalid' read clusters successfully validated from at least one assay. Finally, we assessed the sensitivity of liWGS to capture high-confidence CNVs from CMA [15], observing that liWGS successfully detected 99.0% (1,642/1,659) and 98.5% (802/814) of all deletions and duplications identified by CMA, respectively. Based on these experiments, we estimated a global false discovery rate (FDR) of 10.6% and false negative rate (FNR) of 5.9% for our SV discovery methods. Importantly, it is likely that the reference-based short-read mapping methods used here (liWGS, capture sequencing, and siWGS) share similar error models, which could have underestimated FDR and FNR. Finally, the resolution of liWGS is restricted proportional to the size of the insert, and both sensitivity and specificity for SV detection is thus greatly reduced for rearrangements smaller than ~5 kb (**Additional File 2: Supplemental Note 1**).

Supplemental Results 2: Coding and noncoding functional annotations for all fully resolved SV. Upon overlaying gene annotations onto the SV detected in this study [24], 27.0% (2,995/11,108) of fully resolved SVs were predicted to likely perturb coding sequence of one or more genes, of which approximately one third (35.3%; 1,057/2,995) were predicted to involve two or more genes. Collectively, SVs impacted 4,067 distinct genes in this cohort, of which 73.1% (2,972/4,067) encoded proteins. From these predictions, we estimated each subject's genome to contain 181 genes altered by SV at the resolution of liWGS, of which half (50.8%; 92/181) were computationally predicted to have at least one normal message truncated, resulting in a loss of function (LoF), and 19.3% (35/181) were subject to whole-gene copy gain (CG) of one or more genes. The remaining predictions comprised a variety of other perturbations, including gene retrocopy, translocated exons, and insertions into gene bodies, the functional outcomes of which are less certain than LoF or CG.

We also considered the contribution of SV to noncoding regulatory elements of the genome. Such noncoding regulatory sites are not as well defined as coding loci, and are further confounded by transience and tissue-specificity [25, 26]. These analyses predicted 35.2% (3,910/11,108) of all fully resolved SV to disrupt at least one candidate promoter or enhancer element derived from ENCODE (v3) histone acetylation marks [27]. Despite the frequent proximity of these noncoding regulatory annotations to genes (84.0% within ±20kb of a gene body), a majority (55.4%; 2,166/3,910) of all SV with predicted noncoding regulatory impact had no direct genic perturbation. Next, by mapping all fully resolved SVs onto annotated boundaries between topologically associated domains (TADs) derived from cultured human fibroblasts [28], we identified 192 SVs in this cohort predicted to disrupt one or more TAD boundaries, indicating that a subset of SVs cause restructuring of the local 3D genome architecture, which recent evidence suggests may have relevance to human disease [29-34].

Supplemental Results 3: Genome-wide SV aggregation analyses. We statistically evaluated all positions in the genome for an unexpected aggregation of SV. While most SV were distributed randomly and mostly consistent with the null expectation for relatively rare genomic events approximating a Poisson point process [35], we identified 20 loci enriched for SV at genome-wide significance after correcting for multiple comparisons, 10 of which were specifically enriched for rare (VF<1%) SV in this cohort (**Additional File 2: Supplemental Figure 6** & **Supplemental Tables 4-5**). Several of these loci were consistent with previously described hotspots of recurrent CNVs occurring in large introns, such as 6q26 (*PARK2*), 7q31.1 (*IMMP2L*), 10q21.3 (*CTNNA3*), 15q11.2 (*CYFIP1*), 20p12.1 (*MACROD2*), and 20q12 (*PTPRT*) [36-41]. Of the ten mutational hotspots for rare SV, five (*PARK2*, *IMMP2L*, *CTNNA3*, *CYFIP1*, *PTPRT*) involved genes with some evidence for roles in a broad spectrum of neurological disorders, including autism, schizophrenia, epilepsies, ID, and ADHD [36, 38, 39, 41-43]. No loci met a threshold of genome-wide significance for enrichment specifically of balanced and complex SVs (*i.e.* excluding canonical CNVs; ≥4 SVs per 100 kb window), though two loci (1p35.p2 & 8q11.21) some evidence of possible mutational hotspots specifically prone to large complex and balanced SV (3 SVs per 100kb; $p=3.1 \times 10^{-6}$; corrected q=0.08).

Supplemental Results 4: Cryptic *de novo* SV that may contribute to ASD risk. Although this study was designed to classify the mutational spectrum of SV, the extensive validation studies performed herein did confirm several *de novo* SVs that had not been previously detected by chromosomal microarray (CMA) and whole-exome sequencing (WES) analyses on these same subjects [15]. Two particular examples merit discussion. The first is the gene *VRK1*, which was disrupted by a relatively small 14 kb *de novo* LoF exonic deletion in one subject with ASD in this cohort (**Additional File 2: Supplemental Figure 9**). *VRK1* is involved in chromatin remodeling [13], shares coexpression patterns with known ASD-associated genes [10], has been implicated in neuronal migration, development, and maintenance [11, 12], and was remarkably predicted to be among the most highly connected genes to other genes that are recurrently mutated in ASD [14]. Yet, as our colleagues previously noted, to date there has been "essentially no genetic evidence for [*VRK1*'s] involvement in ASD" [14]. The second example is a 675 kb *de novo* inversion at the 2q23.1 reciprocal microdeletion/microduplication syndrome locus, which encompasses *MBD5*, the syndrome's proposed driver gene (**Additional File 2: Supplemental Figure 10**) [19-21]. This inversion did not directly disrupt *MBD5*, instead truncating a proximal gene, *ACVR2A*, and quantitative real-time PCR follow-up studies in available lymphoblastoid cell lines (LCLs) revealed reduced expression of *ACVR2A*, but not *MBD5*, commensurate with a heterozygous LoF mutation. Both *MBD5* and *ACVR2A* are constrained against LoF mutations in healthy individuals and are within in the 2q23.1 syndrome critical region [17-21], suggesting this *de novo* inversion may provide an initial clue for a possible secondary contributing locus in the 2q23.1 microdeletion syndrome, although a more concrete interpretation is challenging due to the complex and as-of-yet unresolved pathogenicity of the 2q23.1 region. These results emphasize the value of capturing the complete spectrum of genomic variation in studies of human disease.

Supplemental Note 1. Definition and validation of cxSVs. In this study, we defined rearrangements as complex if the variant allele structure involved two or more distinct rearrangement signatures (*e.g.* inversion & deletion) or if the rearrangement involved at least three breakpoints detectable at the resolution of liWGS; this breakpoint resolution varies by coverage and by genomic locus, but is approximately 1.5kb in most cases. However, as has been highlighted by several studies [4, 44-47], SV breakpoints frequently do not form precise junctions on the derivative chromosomes, and instead involve micro-inversions, small non-templated insertions, or other micro-complexity. As liWGS does not provide nucleotide-level resolution of breakpoints, we were unable to categorically assess any additional breakpoint complexity below ~1.5kb. A previous study sequenced the breakpoints of 38 cxSVs detected by liWGS, finding that 5/38 (13%) rearrangements had complexity at one or more breakpoints below the detection thresholds of liWGS [48]; despite small sample sizes, these prior data provide a rough estimate of the potential frequency of SV complexity not captured by liWGS in the present study. Thus, there likely is a meaningful subset of SV that are misclassified in this present study as a consequence of the relatively limited resolution of liWGS, and that the sixteen cxSV subclasses delineated here are almost certainly an underrepresentation of the total complement of cxSVs existent in the human genome.

Supplemental References

1. Neale BM, Kou Y, Liu L, Ma'ayan A, Samocha KE, Sabo A, Lin CF, Stevens C, Wang LS, Makarov V, et al: **Patterns and rates of exonic de novo mutations in autism spectrum disorders.** *Nature* 2012, **485:**242-245.
2. De Rubeis S, He X, Goldberg AP, Poultney CS, Samocha K, Cicek AE, Kou Y, Liu L, Fromer M, Walker S, et al: **Synaptic, transcriptional and chromatin genes disrupted in autism.** *Nature* 2014, **515:**209-215.
3. Collins RL, Stone MR, Brand H, Glessner JT, Talkowski ME: **CNView: a visualization and annotation tool for copy number variation from whole-genome sequencing.** *bioRxiv* 2016.
4. Sudmant PH, Rausch T, Gardner EJ, Handsaker RE, Abyzov A, Huddleston J, Zhang Y, Ye K, Jun G, Hsi-Yang Fritz M, et al: **An integrated map of structural variation in 2,504 human genomes.** *Nature* 2015, **526:**75-81.
5. Zheng GX, Lau BT, Schnall-Levin M, Jarosz M, Bell JM, Hindson CM, Kyriazopoulou-Panagiotopoulou S, Masquelier DA, Merrill L, Terry JM, et al: **Haplotyping germline and cancer genomes with high-throughput linked-read sequencing.** *Nat Biotechnol* 2016.
6. Liu P, Erez A, Nagamani SC, Dhar SU, Kolodziejska KE, Dharmadhikari AV, Cooper ML, Wiszniewska J, Zhang F, Withers MA, et al: **Chromosome catastrophes involve replication mechanisms generating complex genomic rearrangements.** *Cell* 2011, **146:**889-903.
7. Stephens PJ, Greenman CD, Fu B, Yang F, Bignell GR, Mudie LJ, Pleasance ED, Lau KW, Beare D, Stebbings LA, et al: **Massive genomic rearrangement acquired in a single catastrophic event during cancer development.** *Cell* 2011, **144:**27-40.
8. Crasta K, Ganem NJ, Dagher R, Lantermann AB, Ivanova EV, Pan Y, Nezi L, Protopopov A, Chowdhury D, Pellman D: **DNA breaks and chromosome pulverization from errors in mitosis.** *Nature* 2012, **482:**53-58.
9. Zhang CZ, Spektor A, Cornils H, Francis JM, Jackson EK, Liu S, Meyerson M, Pellman D: **Chromothripsis from DNA damage in micronuclei.** *Nature* 2015, **522:**179-184.
10. Parikshak NN, Luo R, Zhang A, Won H, Lowe JK, Chandran V, Horvath S, Geschwind DH: **Integrative functional genomic analyses implicate specific molecular pathways and circuits in autism.** *Cell* 2013, **155:**1008-1021.
11. Renbaum P, Kellerman E, Jaron R, Geiger D, Segel R, Lee M, King MC, Levy-Lahad E: **Spinal muscular atrophy with pontocerebellar hypoplasia is caused by a mutation in the VRK1 gene.** *Am J Hum Genet* 2009, **85:**281-289.
12. Vinograd-Byk H, Sapir T, Cantarero L, Lazo PA, Zeligson S, Lev D, Lerman-Sagie T, Renbaum P, Reiner O, Levy-Lahad E: **The spinal muscular atrophy with pontocerebellar hypoplasia gene VRK1 regulates neuronal migration through an amyloid-beta precursor protein-dependent mechanism.** *J Neurosci* 2015, **35:**936-942.
13. Salzano M, Sanz-Garcia M, Monsalve DM, Moura DS, Lazo PA: **VRK1 chromatin kinase phosphorylates H2AX and is required for foci formation induced by DNA damage.** *Epigenetics* 2015, **10:**373-383.
14. Liu L, Lei J, Sanders SJ, Willsey AJ, Kou Y, Cicek AE, Klei L, Lu C, He X, Li M, et al: **DAWN: a framework to identify autism genes and subnetworks using gene expression and genetics.** *Mol Autism* 2014, **5:**22.
15. Sanders SJ, He X, Willsey AJ, Ercan-Sencicek AG, Samocha KE, Cicek AE, Murtha MT, Bal VH, Bishop SL, Dong S, et al: **Insights into Autism Spectrum Disorder Genomic Architecture and Biology from 71 Risk Loci.** *Neuron* 2015, **87:**1215-1233.
16. Iossifov I, O'Roak BJ, Sanders SJ, Ronemus M, Krumm N, Levy D, Stessman HA, Witherspoon KT, Vives L, Patterson KE, et al: **The contribution of de novo coding mutations to autism spectrum disorder.** *Nature* 2014, **515:**216-221.
17. Samocha KE, Robinson EB, Sanders SJ, Stevens C, Sabo A, McGrath LM, Kosmicki JA, Rehnstrom K, Mallick S, Kirby A, et al: **A framework for the interpretation of de novo mutation in human disease.** *Nat Genet* 2014, **46:**944-950.
18. Lek M, Karczewski KJ, Minikel EV, Samocha KE, Banks E, Fennell T, O'Donnell-Luria AH, Ware JS, Hill AJ, Cummings BB, et al: **Analysis of protein-coding genetic variation in 60,706 humans.** *Nature* 2016, **536:**285-291.

19. Talkowski ME, Mullegama SV, Rosenfeld JA, van Bon BW, Shen Y, Repnikova EA, Gastier-Foster J, Thrush DL, Kathiresan S, Ruderfer DM, et al: **Assessment of 2q23.1 microdeletion syndrome implicates MBD5 as a single causal locus of intellectual disability, epilepsy, and autism spectrum disorder.** *Am J Hum Genet* 2011, **89:**551-563.
20. Hodge JC, Mitchell E, Pillalamarri V, Toler TL, Bartel F, Kearney HM, Zou YS, Tan WH, Hanscom C, Kirmani S, et al: **Disruption of MBD5 contributes to a spectrum of psychopathology and neurodevelopmental abnormalities.** *Mol Psychiatry* 2014, **19:**368-379.
21. Mullegama SV, Rosenfeld JA, Orellana C, van Bon BW, Halbach S, Repnikova EA, Brick L, Li C, Dupuis L, Rosello M, et al: **Reciprocal deletion and duplication at 2q23.1 indicates a role for MBD5 in autism spectrum disorder.** *Eur J Hum Genet* 2014, **22:**57-63.
22. Sanders SJ, Ercan-Sencicek AG, Hus V, Luo R, Murtha MT, Moreno-De-Luca D, Chu SH, Moreau MP, Gupta AR, Thomson SA, et al: **Multiple recurrent de novo CNVs, including duplications of the 7q11.23 Williams syndrome region, are strongly associated with autism.** *Neuron* 2011, **70:**863-885.
23. Turner TN, Hormozdiari F, Duyzend MH, McClymont SA, Hook PW, Iossifov I, Raja A, Baker C, Hoekzema K, Stessman HA, et al: **Genome Sequencing of Autism-Affected Families Reveals Disruption of Putative Noncoding Regulatory DNA.** *Am J Hum Genet* 2016, **98:**58-74.
24. Rosenbloom KR, Armstrong J, Barber GP, Casper J, Clawson H, Diekhans M, Dreszer TR, Fujita PA, Guruvadoo L, Haeussler M, et al: **The UCSC Genome Browser database: 2015 update.** *Nucleic Acids Res* 2015, **43:**D670-681.
25. Ong CT, Corces VG: **Enhancer function: new insights into the regulation of tissue-specific gene expression.** *Nat Rev Genet* 2011, **12:**283-293.
26. Visel A, Blow MJ, Li Z, Zhang T, Akiyama JA, Holt A, Plajzer-Frick I, Shoukry M, Wright C, Chen F, et al: **ChIP-seq accurately predicts tissue-specific activity of enhancers.** *Nature* 2009, **457:**854-858.
27. Thurman RE, Rynes E, Humbert R, Vierstra J, Maurano MT, Haugen E, Sheffield NC, Stergachis AB, Wang H, Vernot B, et al: **The accessible chromatin landscape of the human genome.** *Nature* 2012, **489:**75-82.
28. Dixon JR, Selvaraj S, Yue F, Kim A, Li Y, Shen Y, Hu M, Liu JS, Ren B: **Topological domains in mammalian genomes identified by analysis of chromatin interactions.** *Nature* 2012, **485:**376-380.
29. Claussnitzer M, Dankel SN, Kim KH, Quon G, Meuleman W, Haugen C, Glunk V, Sousa IS, Beaudry JL, Puviindran V, et al: **FTO Obesity Variant Circuitry and Adipocyte Browning in Humans.** *N Engl J Med* 2015, **373:**895-907.
30. Horn S, Figl A, Rachakonda PS, Fischer C, Sucker A, Gast A, Kadel S, Moll I, Nagore E, Hemminki K, et al: **TERT promoter mutations in familial and sporadic melanoma.** *Science* 2013, **339:**959-961.
31. Lupianez DG, Kraft K, Heinrich V, Krawitz P, Brancati F, Klopocki E, Horn D, Kayserili H, Opitz JM, Laxova R, et al: **Disruptions of topological chromatin domains cause pathogenic rewiring of gene-enhancer interactions.** *Cell* 2015, **161:**1012-1025.
32. Lupianez DG, Spielmann M, Mundlos S: **Breaking TADs: How Alterations of Chromatin Domains Result in Disease.** *Trends Genet* 2016, **32:**225-237.
33. Ordulu Z, Kammin T, Brand H, Pillalamarri V, Redin CE, Collins RL, Blumenthal I, Hanscom C, Pereira S, Bradley I, et al: **Structural Chromosomal Rearrangements Require Nucleotide-Level Resolution: Lessons from Next-Generation Sequencing in Prenatal Diagnosis.** *Am J Hum Genet* 2016, **99:**1015-1033.
34. Redin C, Brand H, Collins RL, Kammin T, Mitchell E, Hodge JC, Hanscom C, Pillalamarri V, Seabra CM, Abbott MA, et al: **The genomic landscape of balanced cytogenetic abnormalities associated with human congenital anomalies.** *Nat Genet* 2016.
35. Lander ES, Waterman MS: **Genomic mapping by fingerprinting random clones: a mathematical analysis.** *Genomics* 1988, **2:**231-239.
36. Ambroziak W, Koziorowski D, Duszyc K, Gorka-Skoczylas P, Potulska-Chromik A, Slawek J, Hoffman-Zacharska D: **Genomic instability in the PARK2 locus is associated with Parkinson's disease.** *J Appl Genet* 2015, **56:**451-461.

37. Bradley WE, Raelson JV, Dubois DY, Godin E, Fournier H, Prive C, Allard R, Pinchuk V, Lapalme M, Paulussen RJ, Belouchi A: **Hotspots of large rare deletions in the human genome.** *PLoS One* 2010, **5:**e9401.
38. Girirajan S, Brkanac Z, Coe BP, Baker C, Vives L, Vu TH, Shafer N, Bernier R, Ferrero GB, Silengo M, et al: **Relative burden of large CNVs on a range of neurodevelopmental phenotypes.** *PLoS Genet* 2011, **7:**e1002334.
39. Lal D, Ruppert AK, Trucks H, Schulz H, de Kovel CG, Kasteleijn-Nolst Trenite D, Sonsma AC, Koeleman BP, Lindhout D, Weber YG, et al: **Burden analysis of rare microdeletions suggests a strong impact of neurodevelopmental genes in genetic generalised epilepsies.** *PLoS Genet* 2015, **11:**e1005226.
40. Mitsui J, Takahashi Y, Goto J, Tomiyama H, Ishikawa S, Yoshino H, Minami N, Smith DI, Lesage S, Aburatani H, et al: **Mechanisms of genomic instabilities underlying two common fragile-site-associated loci, PARK2 and DMD, in germ cell and cancer cell lines.** *Am J Hum Genet* 2010, **87:**75-89.
41. Zhao Q, Li T, Zhao X, Huang K, Wang T, Li Z, Ji J, Zeng Z, Zhang Z, Li K, et al: **Rare CNVs and tag SNPs at 15q11.2 are associated with schizophrenia in the Han Chinese population.** *Schizophr Bull* 2013, **39:**712-719.
42. Schuurs-Hoeijmakers JH, Vulto-van Silfhout AT, Vissers LE, van de V, II, van Bon BW, de Ligt J, Gilissen C, Hehir-Kwa JY, Neveling K, del Rosario M, et al: **Identification of pathogenic gene variants in small families with intellectually disabled siblings by exome sequencing.** *J Med Genet* 2013, **50:**802-811.
43. Glessner JT, Wang K, Cai G, Korvatska O, Kim CE, Wood S, Zhang H, Estes A, Brune CW, Bradfield JP, et al: **Autism genome-wide copy number variation reveals ubiquitin and neuronal genes.** *Nature* 2009, **459:**569-573.
44. Abyzov A, Li S, Kim DR, Mohiyuddin M, Stutz AM, Parrish NF, Mu XJ, Clark W, Chen K, Hurles M, et al: **Analysis of deletion breakpoints from 1,092 humans reveals details of mutation mechanisms.** *Nat Commun* 2015, **6:**7256.
45. Conrad DF, Bird C, Blackburne B, Lindsay S, Mamanova L, Lee C, Turner DJ, Hurles ME: **Mutation spectrum revealed by breakpoint sequencing of human germline CNVs.** *Nat Genet* 2010, **42:**385-391.
46. Carvalho CM, Ramocki MB, Pehlivan D, Franco LM, Gonzaga-Jauregui C, Fang P, McCall A, Pivnick EK, Hines-Dowell S, Seaver LH, et al: **Inverted genomic segments and complex triplication rearrangements are mediated by inverted repeats in the human genome.** *Nat Genet* 2011, **43:**1074-1081.
47. Chiang C, Jacobsen JC, Ernst C, Hanscom C, Heilbut A, Blumenthal I, Mills RE, Kirby A, Lindgren AM, Rudiger SR, et al: **Complex reorganization and predominant non-homologous repair following chromosomal breakage in karyotypically balanced germline rearrangements and transgenic integration.** *Nat Genet* 2012, **44:**390-397, S391.
48. Brand H, Collins RL, Hanscom C, Rosenfeld JA, Pillalamarri V, Stone MR, Kelley F, Mason T, Margolin L, Eggert S, et al: **Paired-Duplication Signatures Mark Cryptic Inversions and Other Complex Structural Variation.** *Am J Hum Genet* 2015, **97:**170-176.
